# Hypermethylation of Auxin-Responsive Motifs in the Promoters of the Transcription Factor Genes Accompanies the Somatic Embryogenesis Induction in Arabidopsis

**DOI:** 10.3390/ijms21186849

**Published:** 2020-09-18

**Authors:** Daria Grzybkowska, Katarzyna Nowak, Małgorzata D. Gaj

**Affiliations:** Faculty of Natural Sciences, Institute of Biology, Biotechnology and Environmental Protection, University of Silesia in Katowice, Jagiellońska 28, 40-032 Katowice, Poland; dariagrzybkowska@interia.pl (D.G.); katarzyna.nowak@us.edu.pl (K.N.)

**Keywords:** DNA methylation, somatic embryogenesis, SE-related genes

## Abstract

The auxin-induced embryogenic reprogramming of plant somatic cells is associated with extensive modulation of the gene expression in which epigenetic modifications, including DNA methylation, seem to play a crucial role. However, the function of DNA methylation, including the role of auxin in epigenetic regulation of the SE-controlling genes, remains poorly understood. Hence, in the present study, we analysed the expression and methylation of the *TF* genes that play a critical regulatory role during SE induction (*LEC1*, *LEC2*, *BBM*, *WUS* and *AGL15*) in auxin-treated explants of Arabidopsis. The results showed that auxin treatment substantially affected both the expression and methylation patterns of the SE-involved *TF* genes in a concentration-dependent manner. The auxin treatment differentially modulated the methylation of the promoter (P) and gene body (GB) sequences of the SE-involved genes. Relevantly, the SE-effective auxin treatment (5.0 µM of 2,4-D) was associated with the stable hypermethylation of the P regions of the SE-involved genes and a significantly higher methylation of the P than the GB fragments was a characteristic feature of the embryogenic culture. The presence of auxin-responsive (AuxRE) motifs in the hypermethylated P regions suggests that auxin might substantially contribute to the DNA methylation-mediated control of the SE-involved genes.

## 1. Introduction

Plants have a unique capacity for switching on of embryogenic development in in vitro cultured somatic cells. Following a process called somatic embryogenesis (SE), embryo-like structures that are capable of regenerating plants are formed. The genes that control the induction of SE have been intensively studied in order to understand the molecular determinants of cellular toti-/pluripotency in plants [1]. In particular, studies on SE in the model plant Arabidopsis have contributed substantially to deciphering the genetic factors that are involved in the embryogenic reprogramming of plant somatic cells [2,3]. In Arabidopsis, similar to other plants, auxin treatment is the most effective method of SE induction [4]. The impact of auxin treatment on the global methylome of in vitro cultured plant explants, including those undergoing SE induction, has been reported [5,6,7]. Moreover, auxin-modulated changes in the global transcriptomes have also been reported in embryogenic cultures of plants including Arabidopsis [8,9,10]. The analysis of global methylome implies that auxin treatment might control the embryogenic reprogramming of explant cells via the DNA methylation-mediated regulation of gene expression [11].

Numerous genes that encode the transcription factors (*TFs*) of auxin/hormone- and stress-related including *LEAFY COTYLEDON* (*LEC1* and *LEC2*), *BABY BOOM* (*BBM*), *AGAMOUS-LIKE15* (*AGL15*) and *WUSCHEL* (*WUS*), which plays a central role in the regulatory network that controls SE induction, had a modulated level of expression during SE induction [2,3]. The LEC1 gene encodes the CCAAT box-binding factor HAP3 subunit, while LEC2 belongs to the LAFL B3 subfamily of the plant-specific B3 domain of the *TFs* [12]. The essential regulatory functions of *LEC1* and *LEC2* in both somatic and zygotic embryogenesis have also been demonstrated [13,14]. The hormone-related functions of LEC1 and LEC2 include the activation of auxin biosynthesis *YUCCA* genes [15,16] and the *LEC2*-mediated control of the GA/ABA balance via feedback regulatory interactions with AGL15 from MADS-box family of *TFs* [17]. The *BBM* gene encodes an AINTEGUMENTA-LIKE (AIL) APETALA2/ethylene-responsive element–binding factor (AP2/ERF) that transcriptionally regulates *LEC1* and *LEC2* during SE induction [18]. The AGL15 contributes to SE induction by controlling the metabolism of gibberellic acid and ethylene and targeting the regulators of auxin signalling [19]. A hormone-related function of the *WUS* gene, a member of the plant-specific homeobox superfamily of the *WOX* genes, was attributed to controlling the cytokinin signalling in stem cells and the regulatory interactions between *WUS* and *LEC2* during SE have also been postulated [20,21].

In conjunction with genetic factors, epigenetic modifications of DNA and histones are believed to play a pivotal role in controlling the developmental processes in animals and plants (reviewed in Reference [22,23]). In Eukaryotes, DNA methylation involves the addition of a methyl group (CH_3_) to the carbon-5 of cytosine in the dinucleotide CpG context. In plants, the CpNpG and CpNpN (N-any nucleotide except for G) sequences are also extensively methylated in DNA [24]. The methylation of cytosine in DNA positively impacts on the compactness of chromatin and, thus, CpG methylation is believed to negatively regulate the gene transcription level [25]. However, the effect of DNA methylation on gene expression depends on the type of the methylated genic sequence, and the methylation of the regulatory and coding sequences of genes seems to impact on gene expression differently [26,27,28].

Changes in promoter methylation have been demonstrated to control the expression of genes in plant development, but the reports have shown diverse and contrasting effects of promoter methylation on gene expression [29,30,31]. Although, the classical concept considers a repressive function of promoter methylation in gene transcription [28,32], a positive effect of promoter methylation on gene expression has also been demonstrated including in plants [33,34]. Moreover, the effect of promoter methylation on gene expression seems to be highly specific to the organ and tissue type [35,36].

Besides promoters, the coding region of gene, termed as “gene body” (GB), might be methylated, and a variety of factors appear to modulate the functional outcome of GB methylation [37,38,39]. A number of reports have provided arguments for the genomic functionality of GB methylation in the ontogenesis and evolution of eukaryotic organisms including plants [25].

The role of DNA methylation in controlling developmental processes *in planta* has been demonstrated [40,41]. The impact of DNA methylation on somatic cell reprogramming in plants has mostly been focused on the effects of a demethylation agent, 5-azacitidine (5-AzaC), on the morphogenic responses of in vitro cultured tissue [42]. The reports have presented conflicting results, and either a positive [43,44,45] or negative [6,46,47] impact of the 5-AzaC treatment on the embryogenic response of the plant explants have been indicated. Both an increased and decreased global DNA methylation level have consistently been reported as accompanying SE induction [6,43,46,48].

Because embryogenic and non-embryogenic cultures can display similar changes in global DNA methylation [6,49], insight into the methylation of specific genes might be more conclusive for revealing the role of this epigenetic mark in plant cell reprogramming in vitro. However, reports on the methylation of specific genes during SE induction are still quite limited. The activation of some hormone-related and *WOX* genes in an embryogenic culture of cotton was associated with decreased methylation of the CHH sites in the gene promoters [50]. The hypomethylation of the promoters of *LEC1* has been reported in an embryogenic culture of *Daucus carota* [51] and, similarly, the *SOMATIC EMBRYOGENESIS RECEPTOR-LIKE KINASE* (*SERK*), *LEC2* and *WUS* genes have been shown to have a decreased methylation level in the embryogenic calli of *Boesenbergia rotunda* [37]. Although the reports suggest that gene methylation might be involved in the embryogenic response of somatic plant cells, more detailed studies are needed in order to reveal the SE-associated relationships between methylation and the expression of the genes that control embryogenic induction.

In a methylome analysis, 5-mC abundance can be evaluated at the global and/or ”site-specific” levels, and a variety of analytical methods are used that differ in their sensitivity and accuracy in detecting 5-mC [52]. The assays that are used to analyse the DNA methylation of the specific genes/regions of interest are based on different techniques, including bisulfite conversion, selective digestion of DNA by specific endonucleases and enrichment strategies in which the methylated DNA is immunoprecipitated with anti-methylcytosine binding proteins (MBD) or antibodies against 5-mC (MeDIP, methylated DNA immunoprecipitation) [52,53].

Here, we used the MeDIP method to gain insights into the role of DNA methylation in the epigenetic regulation of SE. To this end, we analysed the changes in the methylation and expression of the transcription factor genes that are associated with SE induction in the model plant Arabidopsis. Arabidopsis explants were cultured in vitro on media with different 2,4-D concentrations, and the level of 5-mC in specific regions of the SE-regulatory genes, including the promoter and gene body sequences, were subjected to a MeDIP analysis. The results indicated that 2,4-D affected both the methylation and expression of the SE-regulatory *TF* genes in a concentration-dependent manner. The SE-effective auxin treatment caused a specific expression pattern of the SE-involved genes that was associated with distinct changes in the methylation status of the auxin-responsive motifs in the gene promoters. The study provides new pieces of evidence on the role of DNA methylation in the epigenetic control of the auxin-mediated embryogenic transition in plant somatic cells.

## 2. Results

### 2.1. 2,4-D Significantly Modulates the Expression Level of the SE-Involved TF Genes in a Concentration-Dependent Manner

Embryogenic transition is associated with the auxin-induced modulation of the explant transcriptome, and the efficiency of SE induction depends on the 2,4-D concentration that is used to treat explants [4]. Hence, we assumed that 2,4-D might affect the gene expression in a concentration-dependent manner. To verify this assumption, we examined the expression profiles of the SE-regulatory genes (*LEC1*, *LEC2*, *BBM*, *WUS* and *AGL15*) in Arabidopsis explants that had been induced on media with different 2,4-D concentrations (0.1, 5.0 and 20.0 µM). The explants that had been cultured on a medium with 5.0 µM of 2,4-D were efficiently induced towards SE, while the other treatments promoted the development of a non-embryogenic callus. The control culture included explants that had been induced on an auxin-free medium (0.0 µM of 2,4-D) that promoted seedling development.

**Figure 1:**
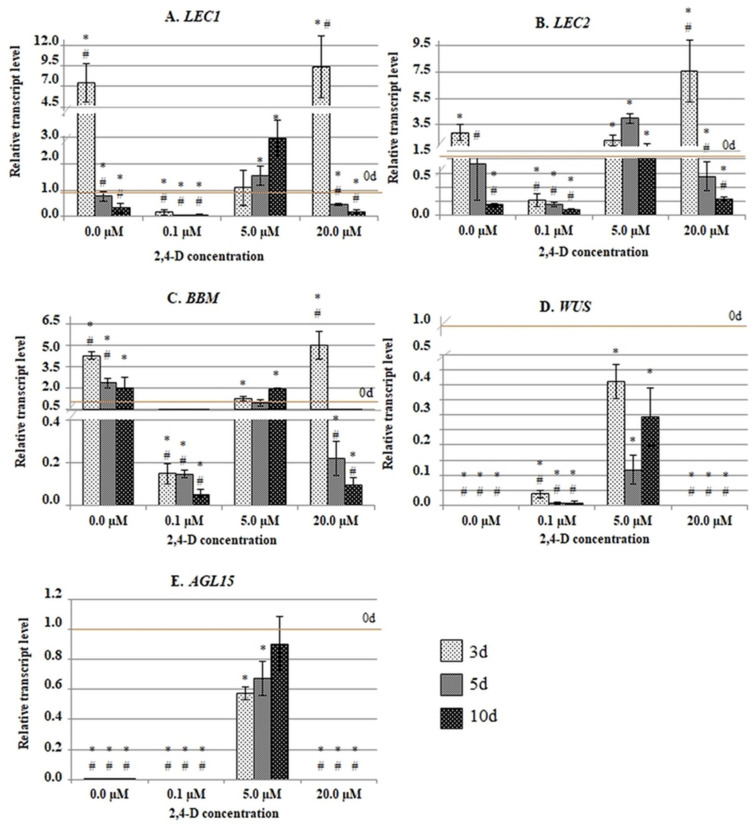
The expression profiles of the SE-involved *TF* genes (**A**–**E**) on media that had been supplemented with different 2,4-D concentrations (0.0 µM; 0.1 µM; 5.0 µM; 20.0 µM). (**A**) *LEC1*, (**B**) *LEC2*, (**C**) *BBM*, (**D**) *WUS* and (**E**) *AGL15*. The relative transcript level was normalised to the internal control (*At4g27090*) and calibrated to the 0 d of culture. Values that were significantly different than 0d are indicated with an asterisk (*);values that were significantly different than 5.0 µM of 2,4-D at the corresponding day of the culture are indicated with a hashtag (#) (Student’s *t*-test, *p* < 0.05). Error bars indicate the SD.

The results of the RT-qPCR analysis revealed two distinct expression profiles of the genes that involve the up- (*LEC1*, *LEC2* and *BBM*) and downregulation (*WUS* and *AGL15*) of gene transcripts in the in vitro cultured explants (Figure 1). Regardless of the general expression profile, the analysed gene responded similarly to the SE-effective treatment with 5.0 µM of 2,4-D. Accordingly, we found that SE induction is associated with a stable and relatively high transcript accumulation compared to the other treatments. It is worth noting that two of the genes, *WUS* and *AGL15*, were expressed exclusively in the explants cultured on the SE-effective medium with 5.0 µM of 2,4-D. By contrast, the treatments that were ineffective in SE induction either resulted in a distinctly lower (0.1 µM of 2,4-D) or transiently increased (20.0 µM of 2,4-D) gene expression.

The analysis indicated that the SE-effective concentration of 2,4-D (5.0 µM) resulted in a specific expression pattern of the *TF* genes. This result implies that the SE-promoting activity of auxin involves a specific transcriptional fine-tuning of the genes that have a regulatory function during SE induction.

### 2.2. Different Methylation of the SE-Involved TF Genes in Response to 2,4-D Treatment

In our previous study, we demonstrated that the global demethylation of DNA by 5-AzaC treatment affected both the induction of SE and the expression of the SE-regulatory genes [6]. Given the specific expression profile of the genes on the SE-effective medium (present results), we assumed that 2,4-D might control the expression of the SE-involved genes in the treated explants via the differential methylation of these genes.

**Figure 2:**
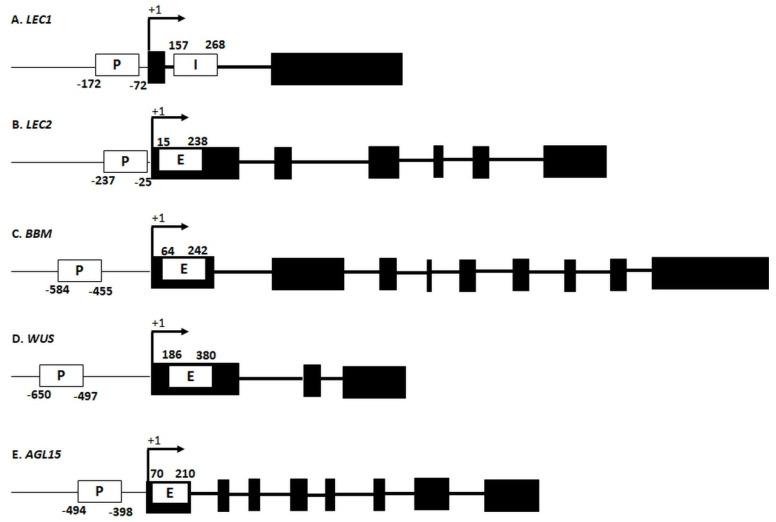
Promotor- and gene body-localised regions of the SE-involved *TF* genes that were analysed using the magMeDIP method. (**A**) *LEC1*, (**B**) *LEC2*, (**C**) *BBM*, (**D**) *WUS* and (**E**) *AGL15*. The exons (black boxes) and introns (thick lines) are indicated. White boxes show the regions within the promoter (P), first exon (E) and intron (I) that were selected for the analysis; the distance of the analysed fragments from the TSS (Transcription Start Site) is indicated.

To verify this hypothesis, changes in the DNA methylation in specific sequences of the SE-regulatory genes were evaluated in explants that had been cultured on media with different 2,4-D concentrations (0.0; 0.1, 5.0 and 20.0 µM). Because the effect of DNA methylation on gene expression might differ among the sequences that have a regulatory or coding function [28], the regions localized in the promoter (P) and gene body (GB) regions of the SE-involved genes were analysed (Table S2; Figure 2). The P fragments of most of the analysed genes (LEC1, *LEC2*, *BBM* and *WUS*) included the ARF-binding auxin-responsive motif (AuxRE). In the promoter of *AGL15*, which lacks the AuxRE, the ethylene response motif was analysed. The analysed GB fragments were localised in the first exon of the genes except for *LEC1* in which a fragment of the first intron was analysed. The results of the magMeDIP analysis are presented in Supplementary Materials Tables S4 and S5 and are described below.

### 2.3. Methylation of the Gene Sequences in Freshly Isolated Explants (0 d)

**Figure 3:**
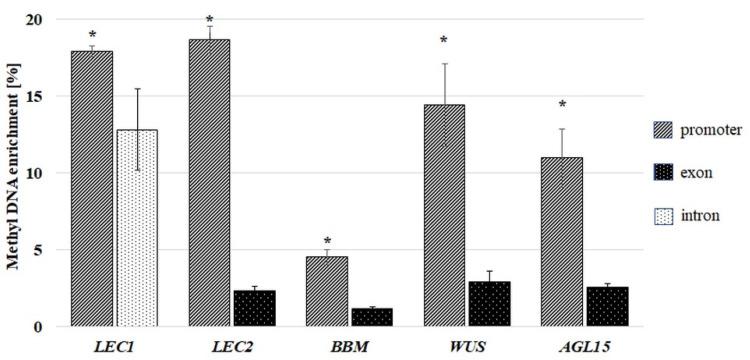
Methyl DNA enrichment (%) in promoter (P) and gene body regions (E and I) of the SE-involved *TF* genes (*LEC1*, *LEC2*, *BBM*, *WUS* and *AGL15*) in the explants before the culture (0 d). Promotor (P), first exon (E) and intron (I) fragment. Any significantly higher methylation of promoter than the gene body fragment of the corresponding gene is indicated with an asterisk (*) (Student’s *t*-test, *p* < 0.05). Error bars indicate the standard deviation (SD).

Analysis of freshly isolated explants (0 d) indicated significant differences in the methylation level between the genes and the types of gene sequences (Figure 3). The P and GB fragments differed markedly in their methylation frequency. On average, 12% and 4% of the P and GB sequences, respectively, were methylated, and the P regions had a distinctly higher (1.5–6.0 fold) frequency of methylation than the GB sequences of the analysed genes. The frequency of the methylated P fragments ranged from 4.5 to 18.8 % depending on the gene, while 1.2 −2.9% of the exonic fragments were methylated. Interestingly, the intron fragment that was analysed in *LEC1* had the highest methylation frequency (12.8%) of the GB fragments.

### 2.4. The Hypomethylation of Both the P and GB Sequences is a Common Response of the SE-Involved Genes to In Vitro Culture Conditions

**Figure 4:**
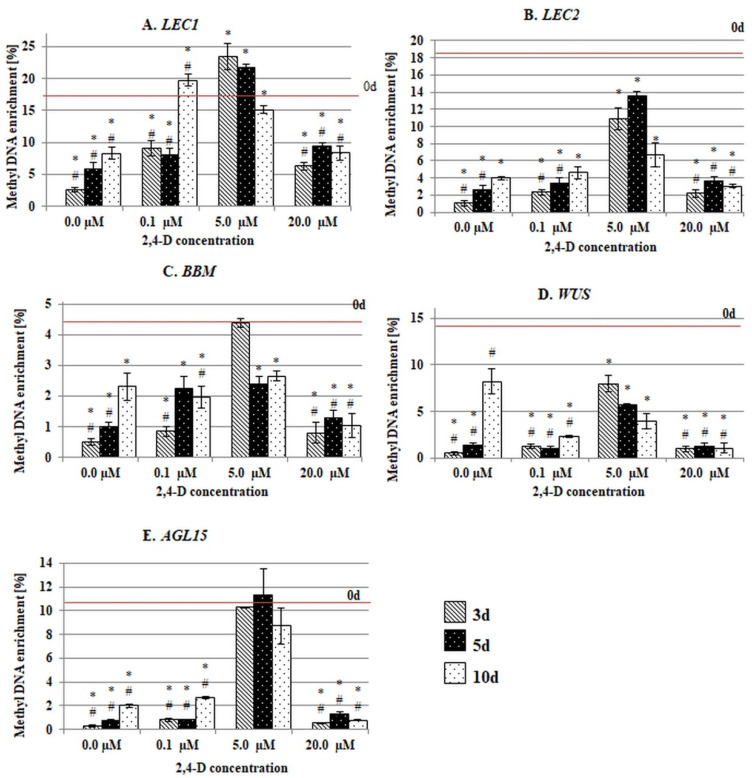
Methyl DNA enrichment (%) in the promotor fragments (P) of the SE-involved *TF* genes (**A**–**E**) in the explants that were cultured on media with different 2,4-D concentrations (0.0 µM; 0.1 µM; 5.0 µM; 20.0 µM). (**A**) *LEC1*, (**B**) *LEC2*, (**C**) *BBM*, (**D**) *WUS* and (**E**) *AGL15*. Values that were significantly different than 0 d are indicated with an asterisk (*). Values that were significantly different than 5.0 µM of 2,4-D at the corresponding day of culture are indicated with a hashtag (#) (Student’s *t*-test, *p* < 0.05). Error bars indicate the standard deviation (SD).

**Figure 5:**
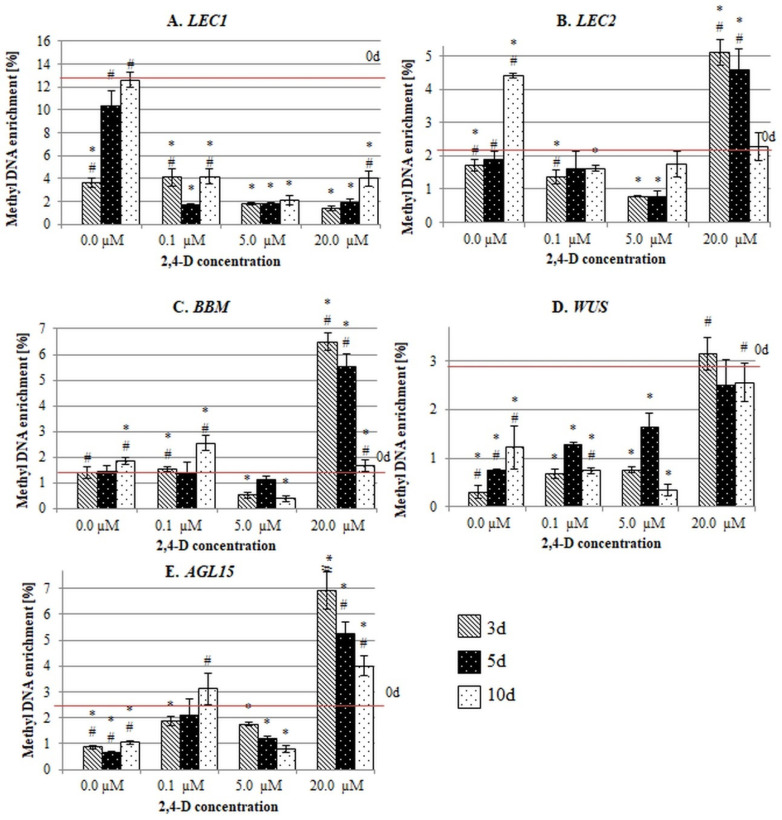
Methyl DNA enrichment in gene body fragments of the SE-involved *TF* genes (**A**–**E**) in the explants that were cultured on media with different 2,4-D concentrations (0.0 µM; 0.1 µM; 5.0 µM; 20.0 µM). (**A**) *LEC1*, (**B**) *LEC2*, (**C**) *BBM*, (**D**) *WUS* and (**E**) *AGL15*. The analysed gene body fragments include the intron (*LEC1*) and exon (*LEC2*, *BBM*, *WUS* and *AGL15*) fragments. Values that were significantly different than 0 d are indicated with an asterisk (*). Values that were significantly different than 5.0 µM of 2,4-D at the corresponding day of the culture are indicated with a hashtag (#) (Student’s *t*-test, *p* < 0.05). Error bars indicate the SD.

After the explants had been transferred to the in vitro conditions, there were significant changes in the methylation of the gene sequences and a considerable decrease in the methylation frequency of genic fragments (both P and GB) in the vast majority of the culture combinations (Figure 4 and Figure 5). It is worth noting that in the majority of the culture combinations, a substantial hypomethylation was characteristic of both the gene fragments that had been treated with 2,4-D and those that were untreated, which suggests that the demethylation genic sequences might manifest a genome response to the abiotic stress that is imposed by in vitro culture conditions.

Despite the similarity of the general response to in vitro culture conditions, there were substantial differences in the methylation frequency in the P and GB sequences relative to the auxin concentration in the medium. Accordingly, the P fragments had the highest methylation frequency in the explants that had been treated with 5.0 µM of 2,4-D that had efficiently induced SE (Figure 4). By contrast, treatment of the explants with 0.1 and 20.0 µM of 2,4-D resulted in a P methylation at a frequency that was similar to that found on the auxin-free medium. This finding suggests that the SE-effective 2,4-D treatment seems to specifically impact on the methylation of the gene promotors.

Conversely, the SE-effective 2,4-D treatment had no specific effect on the methylation of the GB sequences (Figure 5). Rather, we found that the treatment that induced a non-embryogenic callus (20.0 µM of 2,4-D) resulting in a significantly increased methylation of the exonic fragments of the *LEC2*, *BBM*, *WUS* and *AGL15* genes. In contrast to the exonic sequences, the intron fragment that was analysed in *LEC1* had a similar methylation frequency in response to the different 2,4-D treatments. These results imply that auxin treatment might affect the exonic and intronic sequences differently.

**Figure 6:**
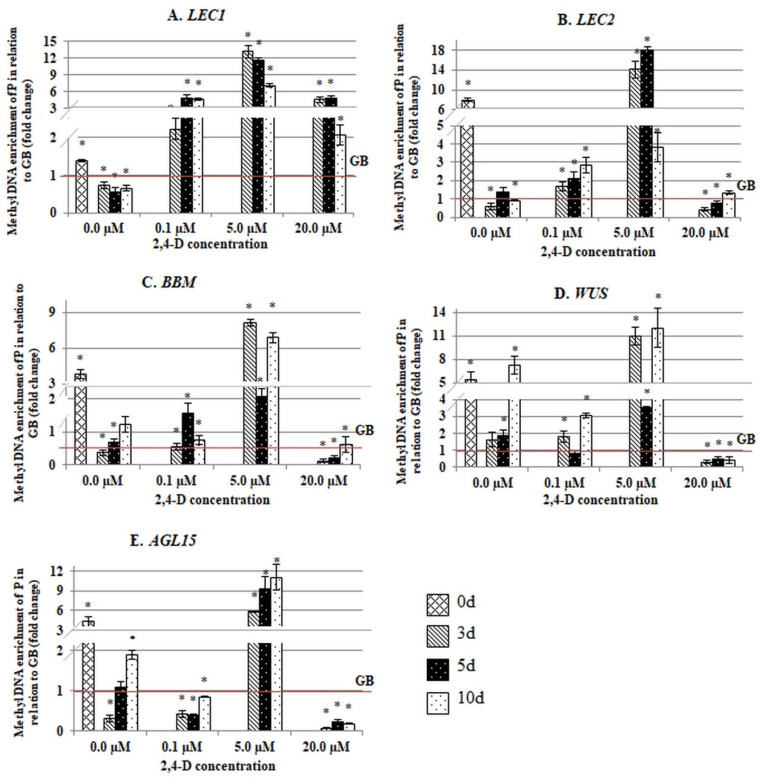
Comparison of the methyl DNA enrichment in the promotor fragments (P) and gene body fragments (GB) of the SE-involved *TF* genes (**A**–**E**) in the explants that were cultured on media with different 2,4-D concentrations (0.0 µM; 0.1 µM; 5.0 µM; 20.0 µM). (**A**) *LEC1*, (**B**) *LEC2*, (**C**) *BBM*, (**D**) *WUS* and (**E**) *AGL15*. The analysed GB fragments included the intron (*LEC1*) and exon (*LEC2*, *BBM*, *WUS* and *AGL15*) fragments. The DNA methylation level of P was calibrated to the GB methylation level of the corresponding gene. Values that were significantly different between the P and GB methylation are indicated with an asterisk (*). (Student’s *t*-test, *p* < 0.05). Error bars indicate the SD.

To expose any differences in the methylation of the P and GB regions, we compared the methylation frequency of the P to GB in the different genes and culture combinations. The analysis confirmed that there was a significantly higher methylation frequency for the P sequences than for the GB sequences in most of the genes and media combinations that were analysed (Figure 6). In particular, the methylation of the P sequences significantly exceeded (from 2 to 17 fold, depending on the gene) that of the GB fragments in the embryogenic culture that had been induced with 5.0 µM of 2,4-D. The genes with opposed expression patterns during SE induction (i.e., the genes of up- (*LEC1*, *LEC2*, *BBM*) and downregulated (*WUS*, *AGL15*) expression in an embryogenic culture) had a high P versus GB ratio of methylation (Figure 1). An inverse methylation ratio between the P to GB regions was observed on the SE-ineffective medium that had been highly enriched in auxin (20.0 µM of 2,4-D) which caused a significant hypermethylation of the exonic sequence. In conclusion, these findings showed that auxin treatment affected the methylation of the regulatory and coding sequences differently and that the SE-effective concentration of 2,4-D (5.0 µM) resulted in the specific hypermethylation of the promoter regions of the SE-involved *TF* genes.

The study provided evidence that the auxin (2,4-D)-triggered mechanism of SE induction might involve the DNA methylation-mediated regulation of the SE-involved *TF* genes. We assumed that the methylation of the specific auxin-related regulatory sequences in the promotors of the *TF* genes was of crucial importance for the correct fine-tuning of the activity of the genes during SE induction.

## 3. Discussion

In our previous study, an inhibition of the embryogenic response and a deregulation of the genes was found in the 5-AzaC-treated culture of Arabidopsis, which suggested a role of DNA methylation in the transcriptional reprogramming of somatic cells during embryogenic induction [6]. Given the essential function of auxin in the SE-induction mechanism [54,55], we assumed that auxin might control the expression of the SE-involved genes by modulating DNA methylation. To verify this assumption, we analysed the association between the changes in methylation and expression of the SE-involved *TF* genes (*LEC1*, *LEC2*, *BBM*, *WUS* and *AGL15*) relative in relevance to the auxin treatment in the in vitro-cultured explants of Arabidopsis.

### 3.1. In Vitro Culture-Related Stress Causes the General Demethylation of the Gene Sequences

We found that a significant hypomethylation of both types of genic sequences, including the regulatory and coding fragments, was a common explant response to an in vitro culture. Similarly, the demethylation of DNA at a global level was reported in in vitro cultured Arabidopsis explants [6]. These findings suggest that the stress that is imposed by in vitro culture conditions, such as isolation and explant transfer to a culture medium, might per se promote the demethylation of DNA [56,57]. Genome-wide changes in the DNA methylation, including DNA hypomethylation, were found to be associated with plant in vitro cultures [47,58,59,60]. Similarly, different biotic and abiotic stresses distinctly modulate the plant methylome at the genome- and gene-level during in vivo development [28,61,62,63,64]. The erasing, at least in part, of the pre-existing epigenetic marks in a genome, including DNA methylation, seems to be required for the developmental transition of cells [65,66,67,68]. Hence, we assumed that the hypomethylation of DNA that is caused by stress, including in vitro culture conditions might prime the somatic cells to become poised for developmental stimuli such as auxin treatment.

### 3.2. The SE-Effective Auxin Concentration Promotes the Methylation of the Promoters of the SE-Regulatory TF Genes

We found that the promoters (P) and coding (GB) sequences of the SE-involved *TF* genes, including *LEC1*, *LEC2*, *BBM*, *WUS* and *AGL15*, showed significantly different methylation patterns in response to 2,4-D, which implies the that there are different roles for P versus GB methylation in the auxin-mediated mechanism that controls SE induction. In line with this assumption, distinct differences in the methylation of different genomic sequences, including the promoters and coding gene regions (exons and introns) have been reported [28,69,70,71], and the various biological function of methylation in the regulatory versus coding sequences was postulated [72,73,74].

The results showed that the methylation of the *TF* genes was distinctly modulated by the 2,4-D treatment in a concentration-dependent manner. In Arabidopsis, treatment of the explants with 2,4-D at a strictly defined concentration (5.0 µM) is required for SE induction, while a decrease or increase of 2,4-D in a medium results in shoot organogenesis or non-embryogenic callus development, respectively [16,75,76,77]. We assumed that the SE-effective auxin treatment might modulate the expression of the SE-involved *TF* genes via the hypermethylation of the promoters specifically. The SE-involved *TFs* seem to belong to a small group of genes (less than 5% in Arabidopsis) of the developmentally regulated methylation of the promoters [69,74]. The analysed *TFs*, similar to other promoter-methylated genes, had a tissue- and organ-specific expression and response to stress [30,39,69,78,79].

The mechanism by which auxin might impact on DNA methylation to control gene expression includes the auxin-mediated regulation of the genes encoding the DNA (de)methylases, which are essential components of the epigenetic mechanism that controls developmental processes [69,80,81,82]. Similarly, the function of the DNA (de)methylases in auxin-induced embryogenic cultures of different plants, including Arabidopsis, has been postulated [6,7,50,83,84,85]. Different DNA methylases and demethylases operate in plant genomes and their sequence-specific functions have been demonstrated [28,86,87,88,89]. Thus, specific enzymes that differentially methylate the promoters of the SE-involved *TF* genes remain to be identified. Recently, auxin was shown to control gene expression by affecting the organisation of chromatin through the TIR1/AFBs auxin-signalling pathway that has been indicated as contributing to the SE-induction mechanism; however, the role of DNA methylation in this regulation remains unclear [90].

The results also showed that the SE-effective auxin treatment seemed to promote the methylation the hormone-related regulatory motifs, including the auxin- and ethylene-responsive regions in the promoters of the SE-involved *TFs*. The methylation of the *cis*-regulatory elements in the gene promoters distinctly changes the capacity of the regulatory proteins for DNA binding and creates new binding sites for the *TFs* [32,91]. Changes in the methylation status of the TF-binding motifs might positively impact the gene expression by recruiting transcriptional activators and preventing the binding of the repressors [28]. Consequently, we hypothesised that the methylation of the AuxRE motifs in the promoters of the *LEC1*, *LEC2*, *BBM* and *WUS* genes might recruit the methylation-sensitive ARFs that have a regulatory function in SE induction [92]. In Arabidopsis, a high number (over 75%) of *TFs* was estimated as being methylation-sensitive, including the ARF5/MONOPTEROS [93], which has been reported as playing role in SE induction [92,93,94,95,96,97,98,99]. Whether the auxin-controlled differential methylation of the *TF* gene promoters affects the binding of auxin-responsive regulatory proteins, including ARF5, to the promoters of the SE-involved genes, requires experimental confirmation.

Auxin has been indicated to modulate the expression of *AGL15* (present results and Reference [100]); however, a lack of the AuxRE motif in the gene promoter suggests an indirect impact of auxin on the *AGL15* expression. Accordingly, auxin might control *AGL15* through auxin-responsive LEC2 and BBM that directly target *AGL15* during SE induction [15,18]. We found that auxin treatment modulated the level of the methylation of the ethylene-responsive element that is present in the *AGL15* promoter, and this implies a contribution of ethylene to the methylation-mediated control of *AGL15* during auxin-induced SE. In support of this scenario, the extensive crosstalk between auxin and ethylene has been documented in plant development [72,101,102,103] including SE induction in Arabidopsis [104]. The molecular components of the auxin–ethylene interactions that control the expression of *AGL15* during SE induction remain to be identified.

Interestingly, AGL15 and several other *TFs* of the SE-regulatory network, including FUS3 (FUSCA3), AIL6/PLT3 (AIL6/PLETHORA3), AIL7/PLT7, PHV (PHAVOLUTA), and CUC (CUP-SHAPED) [18,105,106,107,108,109] were found among the *TFs* that have a methylation-sensitive regulatory activity [110]. Thus, the role of DNA methylation in controlling the genes that are targeted by the methylation-sensitive *TFs* during SE might be explored in future studies.

The results showed that the 2,4-D treatment modulated both the expression and methylation in the SE-involved genes in a concentration-dependent manner. Importantly, the SE-effective auxin treatment (5.0 µM of 2,4-D) promoted both the expression and methylation of the gene promoter regions that control the hormone (auxin) responses, which suggests a role of DNA methylation in the transcriptional control of genes during auxin-induced SE. However, the relationships between the auxin treatment, the methylation of the promoters and the expression of the SE-involved genes did not seem to be straight and obvious for all of the genes (Supplementary Materials Figure S1). Such an association was clear for *WUS* and *AGL15* because the genes were activated exclusively after treatment with the SE-effective auxin concentration, which caused the hypermethylation of the promoters. Thus, *AGL15* and *WUS* seem to be candidates that could be used to study the quantitative relationship between auxin and gene transcription [111].

The results also imply a positive impact of promoter methylation on gene expression in SE induction. Reports have shown highly diverse and contrasting effects of promoter methylation on gene expression in embryogenic cultures of plants. Accordingly, both a negative [26,50,112,113], and a positive [50,79,84,114] impact of promoter methylation on specific gene expression in embryogenic cultures of different plants has been demonstrated. The outcome of promoter methylation on gene expression seems to strongly depend on the content of methylated cytosine and the position of the methylated region in the gene promoter, specifically, the distance from the transcription start site, TSS [50,69,115,116]. Consequently, we found the hypermethylation of the promoter fragments that were located in the proximity of the TSS (up to -172 bp for *LEC1* and -650 bp for *WUS*) to be associated with an increased gene expression in SE. By contrast, the hypomethylation of the regulatory regions that were far from the TSS (more than 2 kbp) was associated with the expression of *LEC1* and *WUS* in an embryogenic culture of *Daucus carota* [51] and shoot-regenerating callus of Arabidopsis [117].

### 3.3. Methylation of the Coding Sequences Seems to Have no Apparent Impact on the Expression of the SE-Involved Genes

In plants, the bodies of highly expressed genes are often extensively methylated, but the relationship between intragenic methylation and this expression is not well established and remains controversial [118,119]. We found that 2,4-D treatment resulted in a differential methylation of the GB sequences in the SE-involved genes. However, the changes in the GB methylation of the SE-involved genes appears to be unspecific for embryogenic induction and might reflect a general response of the GB regions to hormones and stress [39,79,120]. Although in a number of reports, GB methylation positively correlated with gene expression [38,116,121,122], other studies demonstrated a lack of any significant impact of GB methylation on the gene transcription [25,123], including that which was expressed in an in vitro culture [84,124]. Similarly, the present results did not reveal any association between GB methylation and the gene expression pattern of the SE-involved genes. In contrast, there was a negative correlation between the DNA methylation in the CG and CHG contexts in the coding sequence of the *SERK*, *BBM*, *LEC2* and *WUS* genes and the expression levels in an in vitro culture of *Boesenbergia rotunda* [37].

The inconclusive results of studies on the relationships between GB methylation and gene expression might result from the diverse locations of the GB sequences that are analysed within genes since the distance of the methylated region from the TSS significantly modulates the effect of methylation on gene transcription [28,36,79,125,126,127]. A clear inverse association of the first exon methylation with gene expression has been reported in mammalian cells [125,126,128]. In plants, the impact of the first exon methylation on gene transcription is less conclusive [28,69,118] and similarly, the SE-specific expression pattern of genes was not associated with the distinct pattern of the first exon methylation in the recent study. However, we found that two of the genes, *LEC2* and *BBM*, had a significant increase of the transcripts and the hypermethylation of the exonic fragments in response to an auxin concentration that was above the optimal (20.0 µM of 2,4-D). By contrast, the intron region that was analysed in *LEC1* was methylated similarly in response to different auxin concentrations (0.1–20.0 µM of 2,4-D). The differential methylation of introns appears to be less frequent than exon methylation, and in Arabidopsis; two-fold fewer genes undergo intron than exon methylation [22,129]. The negative impact of the first intron methylation on gene transcription was found to be characteristic of the majority of genes in animals [130]. In plants, reports have demonstrated the highly diverse and contrasting effects of intron methylation on gene regulation that seem to be modulated by a variety of factors, such as the presence of transposon and the *cis*-regulatory element in the intron, and the context of the methylated cytosine [131,132,133,134,135,136].

In a few reports, the level of intron versus exon methylation was evaluated, and both similarities and differences in the methylation frequencies of these sequences were observed [127,137]. We noted that the intronic sequence of the *LEC1* gene had a distinctly higher level of methylation compared to the exonic fragments that were analysed in the other SE-involved genes. The question of the functionality of intron methylation in *LEC1* remains open. The methylation of introns is primarily postulated as controlling gene expression by maintaining the heterochromatin marks within the gene in order to silence the transposons [116,138,139] or as affecting the gene splicing [132,140,141]. However, the *LEC1* gene is built of only one intron, which lacks transposon elements.

## 4. Materials and Methods

### 4.1. The Plant Material and Growth Conditions

*Arabidopsis thaliana* (L.) Heynh. plants of the Columbia (Col−0) ecotype (purchased from NASC; The Nottingham Arabidopsis Stock Centre, UK; http://arabidopsis.info/) were used as the source of the explants for the in vitro culture in this study. The plants were grown in a phytotron under controlled conditions at 20–22 °C under a 16 h/8 h (light/dark) photoperiod of 100 µM photons m^−2^ s^−1^ of white, fluorescent light.

### 4.2. The Induction of SE on Different 2,4-D Concentrations and In Vitro Culture Conditions

Immature zygotic embryos (IZEs) at the late cotyledonary stage of development were used as the explants for SE induction [75]. After isolation from the siliques, the explants were cultured in vitro on a solid medium containing B5 salts and vitamins [142], 20 g L^−1^ sucrose and 8 g L^−1^ agar (Oxoid, Hampshire, United Kingdom) and supplemented with different concentrations of 2,4-D (0.0; 0.1 µM; 5.0 µM; 20.0 µM). The plant materials that were grown in sterile conditions were kept at 23 °C under a 16 h photoperiod of 40 µM m^−2^ s^−1^ white, fluorescent light.

Because the induction of embryogenic response in the 2,4-D-treated IZEs in Arabidopsis takes up to 8–10 days [75,76], the explants that had been induced on the culture medium for 0, 5 and 10 days were used for molecular analysis. For RNA/DNA isolation at the selected time points of culture, a corresponding number of explants were used to start a culture: 350–450 for 0 d, 250–350 for 3 d, 5 d and 50–100 for 10 d (d = day of culture) for one biological replicate. All of the DNA/RNA material from the corresponding days of culture were isolated from three biological replicates.

### 4.3. RNA Isolation, RT-PCR and Gene Expression Analysis (RT-qPCR)

The total RNA was isolated using an RNAqueous Total RNA Isolation Kit (Thermo Fisher Scientific, Waltham, MA, USA) from explants that were obtained from 0 d, 3 d, 5 d and 10 d (d = day of culture) cultures with different 2,4-D concentrations. The amount and purity of the isolated RNA were evaluated with an ND−1000 spectrophotometer (Nano Drop technologies, LLC, Wilmington, DE, USA). In order to prevent the potential DNA contamination of RNA, the probes were incubated with DNAze (RQ1 RNase-free DNase I kit; Promega Corporation, Madison, WI, USA). The reverse transcription reaction (RT-PCR) was conducted using a Revert Aid First Strand cDNA Synthesis Kit (Thermo Fisher Scientific, Waltham, MA, USA). All of the described steps from RNA isolation to the cDNA synthesis were performed in accordance with the manufacturer’s instructions for each kit.

To analyse the gene expression level for the selected genes encoding *TFs*, after RT-PCR reaction, the synthetised cDNA was diluted with water at 1:4 rate and a 2.5 μL prepared cDNA solution were used for the RT-qPCR (Real-Time Quantitative PCR) reaction, each sample was analysed in two technical replicates. All of the RT-qPCR reactions were performed using an LC480 Instrument II system (ROCHE, Basel, Switzerland) and LightCycler 480 SYBR Green I Master Mix (ROCHE, Basel, Switzerland). The relative expression level was calculated using 2^−ΔΔCT^ methods and was normalised to an internal control—the *At4g27090* gene encoding the 60 S ribosomal protein [6,16,108,143,144,145,146]. All of the primers for the RT-qPCR analysis are listed in Table S1.

### 4.4. DNA Isolation

The total DNA was extracted from explants that were obtained from 0 d, 3 d, 5 d and 10 d (d = day of culture) cultures with different 2,4-D concentrations using the micro c-TAB methods with some slight modifications that corresponded to the durations of the centrifugations [147].

### 4.5. The magMeDIP (Magnetic Methylated DNA Immunoprecipitation) Technique

The DNA methylation level in selected fragments of genomic DNA was analysed using the magMeDIP technique (magnetic Methylated DNA Immunoprecipitation kit; DIAGENODE; Denville, NJ, USA, which is based on MeDIP methode [53,148]). All of the steps of procedure were performed according to the to manufacturer’s instruction with a slight modification: magMeDIP kit’s internal control (metDNA/unmetDNA from Arabidopsis) were treated as an independent probe next to the tested DNA samples and, therefore, the control DNA was not added to the DNA samples from SE culture and were replaced with water. The immunoprecipitation of all of the DNA probes was performed in two technical replicates and three biological replicates. This technique enables any DNA sequence with at least one methylated cytosine to be identified. The immunoprecipitated DNA fractions were analysed next using RT-qPCR with sequence-specific primers.

### 4.6. The RT-qPCR Analysis of the DNA Methylation Level

Each experimental combination was represented by two samples: (a) a fraction of the methylated DNA and (b) the total, non- and methylated DNA. The efficiency of methyl DNA immunoprecipitation was calculated according to manufacturer’s instruction (DIAGENODE; Denville, NJ, USA): 5-mC (%) = 2^[(Ct(10% input)−3.32)-Ct(met DNA IP)*100%]^ were Ct(10% input)—Ct for the total, non- and methylated DNA (b) and Ct(met DNA IP)—Ct for the methylated DNA (a). All of the RTqPCR reactions were performed in two technical and three biological replicates using the LC480 Instrument II system (ROCHE, Basel, Switzerland) and LightCycler 480 SYBR Green I Master Mix (ROCHE).

### 4.7. The Primers for the RT-qPCR Analysis

Creating the RT-qPCR primers: for the primers dedicated to the promotor fragments, a bioinformatic analysis was conducted and a search of their regulatory motifs was performed. All of the gene encoding sequences were downloaded from the NCBI (NCBI, Bethesda, MD, USA http://www.ncbi.nlm.nih.gov). The regulatory motifs were screened using some open-access databases (softberry; Mount Kisco, NY, USA—http://linux1.softberry.com; AGRISH; Ohio, OH, USA—http://arabidopsis.med.ohio-state.edu; PLANT CARE; Gent, Belgium—http://bioinformatics.psb.ugent.be). Most of the genes that were analysed contained the ARF-binding motif within the promotor regions (*LEC2*, *LEC1*, *WUS* and *BBM*), for one gene (*AGL15*), the ethylene-response element was selected as the main target for creating the primers for RT-qPCR (Table S2).The primers for gene body fragments were created in based on the 1st exons encoding sequences with the exception of the *LEC1* gene in which the sequence that was localised in the 1st intron was analysed. All of the primers that were used in the RT-qPCR reactions are listed in Table S3.

### 4.8. Statistical Analyses

Statistical analysis of any significant differences between the samples from the analysed experiments was performed using Statistica v.12 software (StatSoft Poland; Cracow, Poland, http://www.statsoft.pl) and the Student’s *t*-test (*p* = 0.05).

## 5. Conclusions and Perspectives

We found that the *TF* genes that are involved in the induction of the embryogenic transition of somatic cells in Arabidopsis are differentially methylated in response to auxin treatment. The methylation of the regulatory and coding sequences seems to affect the gene expression differently, and the hypermethylation of the hormone (auxin)-responsive motifs in the gene promoters seems to be involved in the auxin-mediated regulation of the *TF* genes during SE induction.

Understanding of the contribution of DNA methylation to the transcriptional regulation of the genes that control SE induction requires insights into the interactions of DNA methylation with the other epigenetic processes [81,82,149]. In Arabidopsis, several studies have provided support for a self-reinforcing loop between DNA and histone methylation, and the CMT2/CMT3 chromomethylases of DNA were found to contain binding sites for silencing the chromatin mark of H3K9me2 [149]. The interplay between DNA methylation and histone methylation could involve the gene repressive PRC2 (POLYCOMB REPRESSIVE COMPLEX 2) complex that has a histone methyltransferase activity with a reported role in controlling the SE-involved genes [150,151]. Moreover, histone acetylation might cooperate with DNA methylation to control gene expression [152,153]. Because histone acetylation affects SE induction [154], the insights into the interplay between DNA methylation and the acetylation of histones are required to decipher the epigenetic mechanism that controls the embryogenic response.

The mechanism of DNA methylation-mediated gene regulation in SE induction might also involve the miRNAs that have an essential regulatory function in SE induction [155]. Relevantly, the miRNAs that operate at the chromatin level to direct the methylation of specific coding sequences have been identified, including a particular class of longer miRNAs [156]. In Arabidopsis, the methylation of the defined sites in the coding sequences of PHB/PHV genes that play a regulatory role in SE might contribute to the miR165/166-mediated regulation of these genes during embryogenic induction [108,157]. Finally, DNA methylation might also impact the miRNA-regulated gene expression by affecting the biogenesis of miRNAs [158,159,160].

In conclusion, to decipher how DNA methylation regulates genes during the embryogenic transition of somatic cells, the complex interplay among the diverse epigenetic processes that control the SE-regulatory genes in response to auxin needs to be explored.

## Supplementary Materials

The following are available online at https://www.mdpi.com/1422-0067/21/18/6849/s1. Table S1. Primers that were used to analyse gene expression (RTqPCR). Table S2. Characteristics of the regulatory motifs in the promoters of the SE-involved genes that were analysed using the magMeDIP method. The motifs include: the ARF-binding (consensus sequence: TGTCTC) and the ethylene-response-element (consensus sequence: TTTGAAAT) sequences (AGRIS, http://arabidopsis.med.ohio-state.edu/AtcisDB; PLANT CARE, http://bioinformatics.psb.ugent.be; SOFT BERRY, http://linux1.softberry.com/). Table S3. The gene primer sequences that were selected for the magMeDIP analysis. Table S4. Methyl DNA enrichment (%) in the promotor (P) fragments of the SE-involved *TF* genes on different 2,4-D concentrations (0.0 µM; 0.1 µM; 5.0 µM; 20.0 µM). Analysis was performed using the magMeDIP method and the percentage of P fragments with methylated cytosine were evaluated. Values that were significantly different than 0d are indicated with an asterisk (*) (Student’s *t*-test, *p* < 0.05). Table S5. Methyl DNA enrichment (%) in the gene body (GB) fragments of the SE-involved *TF* genes on different 2,4-D concentrations (0.0 µM; 0.1 µM; 5.0 µM; 20.0 µM). Analysis was performed using the magMeDIP method and the percentage of the GB fragments with methylated cytosine were evaluated. Values significantly different from the 0 d are indicated with an asterisk (*) (Student’s *t*-test, *p* < 0.05). Figure S1. Transcript level of the SE-involved *TF* genes (**A**–**E**) relative to the methylation of promoter (P) and the gene body (GB) in explants that were cultured on media with different 2,4-D concentrations (0.0 µM; 0.1 µM; 5.0 µM; 20.0 µM). (**A**) *LEC1*, (**B**) *LEC2*, (**C**) *BBM*, (**D**) *WUS* and (**E**) *AGL15*. The analysed GB fragments include the intron (*LEC1*) and exon (*LEC2*, *BBM*, *WUS* and *AGL15*) regions. Error bars indicate the standard deviation (SD).

## References

[1] Fehér A. (2019). Callus, Dedifferentiation, Tot potency, Somatic Embryogenesis: What These Terms Mean in the Era of Molecular Plant Biology?. Front. Plant. Sci..

[2] Nowak K., Gaj M.D. (2016). Transcription Factors in the Regulation of Somatic Embryogenesis. Somat. Embryog. Fundam. Asp. Appl..

[3] Wójcik A.M., Wójcikowska B., Gaj M.D. (2020). Current perspectives on the auxin-mediated genetic network that controls the induction of somatic embryogenesis in plants. Int. J. Mol. Sci..

[4] Wójcikowska B., Gaj M.D. (2016). Somatic Embryogenesis: Fundamental Aspects and Applications. Somat. Embryog. Arab..

[5] Orłowska R., Machczyńska J., Oleszczuk S., Zimny J., Bednarek P.T. (2016). DNA methylation changes and TE activity induced in tissue cultures of barley (*Hordeum vulgare* L.). J. Biol. Res..

[6] Grzybkowska D., Morończyk J., Wójcikowska B., Gaj M.D. (2018). Azacitidine (5-AzaC)-treatment and mutations in DNA methylase genes affect embryogenic response and expression of the genes that are involved in somatic embryogenesis in Arabidopsis. Plant. Growth Regul..

[7] Ji L., Mathioni S.M., Johnson S., Tucker D., Bewick A.J., Kim K.D., Daron J., Slotkin R.K., Jackson S.A., Parrott W.A. (2019). Genome-wide reinforcement of DNA methylation occurs during somatic embryogenesis in soybean. Plant Cell.

[8] Singla B., Tyagi A.K., Khurana J.P., Khurana P. (2007). Analysis of expression profile of selected genes expressed during auxin-induced somatic embryogenesis in leaf base system of wheat (Triticum aestivum) and their possible interactions. Plant. Mol. Biol..

[9] Yang X., Li L. (2012). Analyzing the microRNA Transcriptome in Plants Using Deep Sequencing Data. Biology.

[10] Gliwicka M., Nowak K., Balazadeh S., Mueller-Roeber B., Gaj M.D. (2013). Extensive Modulation of the Transcription Factor Transcriptome during Somatic Embryogenesis in Arabidopsis thaliana. PLoS ONE.

[11] Chapman E.J., Estelle M. (2009). Mechanism of auxin-regulated gene expression in plants. Annu. Rev. Genet..

[12] Harada J.J. (2001). Role of Arabidopsis LEAFY COTYLEDON genes in seed development. J. Plant. Physiol..

[13] Gaj M.D., Zhang S., Harada J.J., Lemaux P.G. (2005). Leafy cotyledon genes are essential for induction of somatic embryogenesis of Arabidopsis. Planta.

[14] Braybrook S.A., Harada J.J. (2008). LECs go crazy in embryo development. Trends Plant. Sci..

[15] Braybrook S.A., Stone S.L., Park S., Bui A.Q., Le B.H., Fischer R.L., Goldberg R.B., Harada J.J. (2006). Genes directly regulated by leafy cotyledon2 provide insight into the control of embryo maturation and somatic embryogenesis. Proc. Natl. Acad. Sci. USA.

[16] Wójcikowska B., Jaskół K., Gasiorek P., Meus M., Nowak K., Gaj M.D. (2013). Leafy cotyledon2 (LEC2) promotes embryogenic induction in somatic tissues of Arabidopsis, via YUCCA-mediated auxin biosynthesis. Planta.

[17] Wang H., Caruso L.V., Downie A.B., Perry S.E. (2004). The embryo MADS domain protein agamous–like 15 directly regulates expression of a gene encoding an enzyme involved in gibberellin metabolism. Plant Cell.

[18] Horstman A., Li M., Heidmann I., Weemen M., Chen B., Muino J.M., Angenent G.C., Boutiliera K. (2017). The BABY BOOM transcription factor activates the LEC1-ABI3-FUS3-LEC2 network to induce somatic embryogenesis. Plant. Physiol..

[19] Zheng Q., Zheng Y., Ji H., Burnie W., Perry S.E. (2016). Gene Regulation by the AGL15 Transcription Factor Reveals Hormone Interactions in Somatic Embryogenesis. Plant. Physiol.

[20] Sablowski R. (2009). Cytokinin and WUSCHEL tie the knot around plant stem cells. Proc. Natl. Acad. Sci. USA.

[21] Su Y.H., Zhao X.Y., Liu Y.B., Zhang C.L., O’Neill S.D., Zhang X.S. (2009). Auxin-induced WUS expression is essential for embryonic stem cell renewal during somatic embryogenesis in Arabidopsis. Plant J..

[22] Feng S., Jacobsen S.E., Reik W. (2010). Epigenetic reprogramming in plant and animal development. Science.

[23] Lauria M., Rossi V. (2011). Epigenetic control of gene regulation in plants. Biochim. Biophys. Acta Gene Regul. Mech..

[24] Stricker S.H., Götz M. (2018). DNA-methylation: Master or slave of neural fate decisions?. Front. Neurosci..

[25] Bewick A.J., Schmitz R.J. (2017). Gene body DNA methylation in plants. Curr. Opin. Plant. Biol..

[26] Stroud H., Greenberg M.V.C., Feng S., Bernatavichute Y.V., Jacobsen S.E. (2013). Comprehensive analysis of silencing mutants reveals complex regulation of the Arabidopsis methylome. Cell.

[27] Niederhuth C.E., Schmitz R.J. (2017). Putting DNA methylation in context: From genomes to gene expression in plants. Biochim. Biophys. Acta Gene Regul. Mech..

[28] Zhang H., Lang Z., Zhu J.K. (2018). Dynamics and function of DNA methylation in plants. Nat. Rev. Mol. Cell Biol..

[29] Liang D., Zhang Z., Wu H., Huang C., Shuai P., Ye C.Y., Tang S., Wang Y., Yang L., Wang J. (2014). Single-base-resolution methylomes of populus trichocarpa reveal the association between DNA methylation and drought stress. BMC Genet..

[30] Feng S.J., Liu X.S., Tao H., Tan S.K., Chu S.S., Oono Y., Zhang X.D., Chen J., Yang Z.M. (2016). Variation of DNA methylation patterns associated with gene expression in rice (*Oryza sativa*) exposed to cadmium. Plant Cell Environ..

[31] Yaish M.W., Al-Lawati A., Al-Harrasi I., Patankar H.V. (2018). Genome-wide DNA Methylation analysis in response to salinity in the model plant caliph medic (Medicago truncatula). BMC Genom..

[32] Zhu H., Wang G., Qian J. (2016). Transcription factors as readers and effectors of DNA methylation. Nat. Rev. Genet..

[33] Williams B.P., Pignatta D., Henikoff S., Gehring M. (2015). Methylation-Sensitive Expression of a DNA Demethylase Gene Serves as an Epigenetic Rheostat. PLoS Genet..

[34] Moreno-Romero J., Jiang H., Santos-González J., Köhler C. (2016). Parental epigenetic asymmetry of PRC 2-mediated histone modifications in the Arabidopsis endosperm. EMBO J..

[35] An Y.Q.C., Goettel W., Han Q., Bartels A., Liu Z., Xiao W. (2017). Dynamic Changes of Genome-Wide DNA Methylation during Soybean Seed Development. Sci. Rep..

[36] Bhatia H., Khemka N., Jain M., Garg R. (2018). Genome-wide bisulphite-sequencing reveals organ-specific methylation patterns in chickpea. Sci. Rep..

[37] Karim R., Tan Y.S., Singh P., Khalid N., Harikrishna J.A. (2018). Expression and DNA methylation of SERK, BBM, LEC2 and WUS genes in in vitro cultures of *Boesenbergia rotunda* (L.) Mansf. Physiol. Mol. Biol. Plants.

[38] Bewick A.J., Zhang Y., Wendte J.M., Zhang X., Schmitz R.J. (2019). Evolutionary and experimental loss of gene body methylation and its consequence to gene expression. G3 GenesGenomesGenet..

[39] Aceituno F.F., Moseyko N., Rhee S.Y., Gutiérrez R.A. (2008). The rules of gene expression in plants: Organ identity and gene body methylation are key factors for regulation of gene expression in Arabidopsis thaliana. BMC Genom..

[40] Kawakatsu T., Nery J.R., Castanon R., Ecker J.R. (2017). Dynamic DNA methylation reconfiguration during seed development and germination. Genome Biol..

[41] Jullien P.E., Susaki D., Yelagandula R., Higashiyama T., Berger F. (2012). DNA methylation dynamics during sexual reproduction in Arabidopsis thaliana. Curr. Biol..

[42] Wójcikowska B., Wójcik A.M., Gaj M.D. (2020). Epigenetic regulation of auxin-induced somatic embryogenesis in plants. Int. J. Mol. Sci..

[43] Fraga H.P.F., Vieira L.N., Caprestano C.A., Steinmacher D.A., Micke G.A., Spudeit D.A., Pescador R., Guerra M.P. (2012). 5-Azacytidine combined with 2,4-D improves somatic embryogenesis of Acca sellowiana (O. Berg) Burret by means of changes in global DNA methylation levels. Plant. Cell Rep..

[44] Osorio-Montalvo P., Sáenz-Carbonell L., De-la-Peña C. (2018). 5-azacytidine: A promoter of epigenetic changes in the quest to improve plant somatic embryogenesis. Int. J. Mol. Sci..

[45] Pila Quinga L.A., Pacheco de Freitas Fraga H., do Nascimento Vieira L., Guerra M.P. (2017). Epigenetics of long-term somatic embryogenesis in *Theobroma cacao* L.: DNA methylation and recovery of embryogenic potential. Plant. Cell. Tissue Organ. Cult..

[46] Teyssier C., Maury S., Beaufour M., Grondin C., Delaunay A., Le Metté C., Ader K., Cadene M., Label P., Lelu-Walter M.-A. (2014). In search of markers for somatic embryo maturation in hybrid larch (Larix × eurolepis): Global DNA methylation and proteomic analyses. Physiol. Plant.

[47] Nic-Can G.I., López-Torres A., Barredo-Pool F., Wrobel K., Loyola-Vargas V.M., Rojas-Herrera R., De-la-Peña C. (2013). New Insights into Somatic Embryogenesis: Leafy cotyledon1, baby boom1 and wuschel-related homeobox4 are epigenetically regulated in coffea canephora. PLoS ONE.

[48] Kwiatkowska A., Zebrowski J., Oklejewicz B., Czarnik J., Halibart-Puzio J., Wnuk M. (2014). The age-dependent epigenetic and physiological changes in an Arabidopsis T87 cell suspension culture during long-term cultivation. Biochem. Biophys. Res. Commun..

[49] Klimaszewska K., Noceda C., Pelletier G., Label P., Rodriguez R., Lelu-Walter M.A. (2009). Biological characterization of young and aged embryogenic cultures of *Pinus pinaster* (Ait.). Vitr. Cell. Dev. Biol. Plant..

[50] Li J., Wang M., Li Y., Zhang Q., Lindsey K., Daniell H., Jin S., Zhang X. (2019). Multi-omics analyses reveal epigenomics basis for cotton somatic embryogenesis through successive regeneration acclimation process. Plant Biotechnol. J..

[51] Shibukawa T., Yazawa K., Kikuchi A., Kamada H. (2009). Possible involvement of DNA methylation on expression regulation of carrot LEC1 gene in its 5′ -upstream region. Gene.

[52] Kurdyukov S., Bullock M. (2016). DNA methylation analysis: Choosing the right method. Biology.

[53] Hsu H.K., Weng Y.I., Hsu P.Y., Huang T.H.M., Huang Y.W. (2014). Detection of DNA methylation by MeDIP and MBDCap assays: An overview of techniques. Mol. Toxicol. Protoc..

[54] Nic-Can G.I., Loyola-Vargas V.M. (2016). The Role of the Auxins During Somatic Embryogenesis *Somat*. Embryog. Fundam. Asp. Appl..

[55] Yang X., Zhang X., Yuan D., Jin F., Zhang Y., Xu J. (2012). Transcript profiling reveals complex auxin signalling pathway and transcription regulation involved in dedifferentiation and redifferentiation during somatic embryogenesis in cotton. BMC Plant. Biol..

[56] Phillips R.L., Kaeppler S.M., Peschke V.M. (1990). Do We. Progress in Plant Cellular and Molecular Biology. Proc. VIIth Int. Congr. Plant Tissue Cell Cult..

[57] Fehér A. (2015). Somatic embryogenesis stress-induced remodeling of plant cell fate. Biochim. Biophys. Acta Gene Regul. Mech..

[58] Rival A., Ilbert P., Labeyrie A., Torres E., Doulbeau S., Personne A., Dussert S., Beulé T., Durand-Gasselin T., Tregear J.W. (2013). Variations in genomic DNA methylation during the long-term in vitro proliferation of oil palm embryogenic suspension cultures. Plant. Cell Rep..

[59] Heringer A.S., Steinmacher D.A., Fraga H.P.F., Vieira L.N., Ree J.F., Guerra M.P. (2013). Global DNA methylation profiles of somatic embryos of peach palm (Bactris gasipaes Kunth) are influenced by cryoprotectants and droplet-vitrification cryopreservation. Plant. Cell. Tissue Organ. Cult..

[60] Pérez M., Viejo M., LaCuesta M., Toorop P., Cañal M.J. (2015). Epigenetic and hormonal profile during maturation of Quercus Suber, L. somatic embryos. J. Plant Physiol..

[61] Chwialkowska K., Nowakowska U., Mroziewicz A., Szarejko I., Kwasniewski M. (2016). Water-deficiency conditions differently modulate the methylome of roots and leaves in barley (*Hordeum vulgare* L.). J. Exp. Bot..

[62] Pandey G., Sharma N., Sahu P.P., Prasad M. (2016). Chromatin-Based Epigenetic Regulation of Plant Abiotic Stress Response. Curr. Genom..

[63] Fortes A.M., Gallusci P. (2017). Plant stress responses and phenotypic plasticity in the epigenomics era: Perspectives on the grapevine scenario, a model for perennial crop plants. Front. Plant. Sci..

[64] Thiebaut F., Hemerly A.S., Ferreira P.C.G. (2019). A role for epigenetic regulation in the adaptation and stress responses of non-model plants. Front. Plant. Sci..

[65] Jacob Y., Martienssen R.A. (2011). Chromatin reprogramming: Gender equality during arabidopsis germline differentiation. Curr. Biol..

[66] Tang X., Lim M.H., Pelletier J., Tang M., Nguyen V., Keller W.A., Tsang E.W.T., Wang A., Rothstein S.J., Harada J.J. (2012). Synergistic repression of the embryonic programme by set domain group 8 and embryonic flower 2 in Arabidopsis seedlings. J. Exp. Bot..

[67] Kumar S., Kumari R., Sharma V., Sharma V. (2013). Roles, and establishment, maintenance and erasing of the epigenetic cytosine methylation marks in plants. J. Genet..

[68] Xu J., Zhou S., Gong X., Song Y., van Nocker S., Ma F., Guan Q. (2018). Single-base methylome analysis reveals dynamic epigenomic differences associated with water deficit in apple. Plant. Biotechnol. J..

[69] Zhang X., Yazaki J., Sundaresan A., Cokus S., Chan S.W.L., Chen H., Henderson I.R., Shinn P., Pellegrini M., Jacobsen S.E. (2006). Genome-wide High-Resolution Mapping and Functional Analysis of DNA Methylation in Arabidopsis. Cell.

[70] Lister R., O’Malley R.C., Tonti-Filippini J., Gregory B.D., Berry C.C., Millar A.H., Ecker J.R. (2008). Highly Integrated Single-Base Resolution Maps of the Epigenome in Arabidopsis. Cell.

[71] Zemlyanskaya E.V., Omelyanchuk N.A., Ubogoeva E.V., Mironova V.V. (2018). Deciphering auxin-ethylene crosstalk at a systems level. Int. J. Mol. Sci..

[72] Jeltsch A. (2010). Phytogeny of methylomes. Science.

[73] Zemach A., Zilberman D. (2010). Evolution of eukaryotic DNA methylation and the pursuit of safer sex. Curr. Biol..

[74] Takuno S., Gaut B.S. (2012). Body-methylated genes in arabidopsis thaliana are functionally important and evolve slowly. Mol. Biol. Evol..

[75] Gaj M.D. (2001). Direct somatic embryogenesis as a rapid and efficient system for in vitro regeneration of Arabidopsis thaliana. Plant. Cell. Tissue Organ. Cult..

[76] Raghavan V. (2004). Role of 2,4–dichlorophenoxyacetic acid (2,4–D) in somatic embryogenesis on cultured zygotic embryos of Arabidopsis: Cell expansion, cell cycling, and morphogenesis during continuous exposure of embryos to 2,4–D. Am. J. Bot..

[77] Kraut M., Wójcikowska B., Ledwoń A., Gaj M.D. (2011). Immature zygotic embryo cultures of Arabidopsis. Amodel system for molecular studies on morphogenic pathways induced in vitro. Acta Biol. Crac. Ser. Bot..

[78] Song Y., Ji D., Li S., Wang P., Li Q., Xiang F. (2012). The dynamic changes of DNA methylation and histone modifications of salt responsive transcription factor genes in soybean. PLoS ONE.

[79] Rajkumar M.S., Shankar R., Garg R., Jain M. (2019). Role of DNA methylation dynamics in desiccation and salinity stress responses in rice cultivars. bioRxiv.

[80] Henderson I.R., Jacobsen S.E. (2007). Epigenetic inheritance in plants. Nature.

[81] Yamamuro C., Zhu J.-K., Yang Z. (2016). Epigenetic Modifications and Plant Hormone Action. Mol. Plant.

[82] Mateo-Bonmatí E., Casanova-Sáez R., Ljung K. (2019). Epigenetic regulation of auxin homeostasis. Biomolecule.

[83] Yamamoto N., Kobayashi H., Togashi T., Mori Y., Kikuchi K., Kuriyama K., Tokuji Y. (2005). Formation of embryogenic cell clumps from carrot epidermal cells is suppressed by 5-azacytidine, a DNA methylation inhibitor. J. Plant. Physiol..

[84] Vining K., Pomraning K.R., Wilhelm L.J., Ma C., Pellegrini M., Di Y., Mockler T.C., Freitag M., Strauss S.H. (2013). Methylome reorganization during in vitro dedifferentiation and regeneration of *Populus trichocarpa*. BMC Plant. Biol..

[85] Jiang F., Xu X., Liu H., Zhu J. (2015). DRM1 and DRM2 are involved in Arabidopsis callus formation. Plant. Cell Tissue Organ. Cult..

[86] Jacobsen S.E., Sakai H., Finnegan E.J., Cao X., Meyerowitz E.M. (2000). Ectopic hypermethylation of flower-specific genes in Arabidopsis. Curr. Biol..

[87] Niederhuth C.E., Bewick A.J., Ji L., Alabady M.S., Kim K.D., Li Q., Rohr N.A., Rambani A., Burke J.M., Udall J.A. (2016). Widespread natural variation of DNA methylation within angiosperms. Genome Biol..

[88] Ashapkin V.V., Kutueva L.I., Aleksandrushkina N.I., Vanyushin B.F. (2019). Epigenetic regulation of plant gametophyte development. Int. J. Mol. Sci..

[89] Liu P., Nie W.F., Xiong X., Wang Y., Jiang Y., Huang P., Lin X., Qin G., Huang H., Niu Q. (2020). A novel protein complex that regulates active DNA demethylation in Arabidopsis. bioRxiv.

[90] Hasegawa J., Sakamoto T., Fujimoto S., Yamashita T., Suzuki T., Matsunaga S. (2018). Auxin decreases chromatin accessibility through the TIR1/AFBs auxin signaling pathway in proliferative cells. Sci. Rep..

[91] Liu L., Jin G., Zhou X. (2015). Modeling the relationship of epigenetic modifications to transcription factor binding. Nucleic Acids Res..

[92] Wójcikowska B., Gaj M.D. (2017). Expression profiling of AUXIN RESPONSE FACTOR genes during somatic embryogenesis induction in Arabidopsis. Plant. Cell Rep..

[93] Thibaud-Nissen F., Shealy R.T., Khanna A., Vodkin L.O. (2003). Clustering of microarray data reveals transcript patterns associated with somatic embryogenesis in soybean. Plant. Physiol..

[94] Shi X., Zhang C., Liu Q., Zhang Z., Zheng B., Bao M. (2016). De novo comparative transcriptome analysis provides new insights into sucrose induced somatic embryogenesis in camphor tree (*Cinnamomum camphora* L.). BMC Genom..

[95] Indoliya Y., Tiwari P., Chauhan A.S., Goel R., Shri M., Bag S.K., Chakrabarty D. (2016). Decoding regulatory landscape of somatic embryogenesis reveals differential regulatory networks between japonica and indica rice subspecies. Sci. Rep..

[96] Capote T., Usié A., Barbosa P., Ramos M., Morais-Cecílio L., Gonçalves S. (2019). Transcriptome dynamics of cork oak (Quercus suber) somatic embryogenesis reveals active gene players in transcription regulation and phytohormone homeostasis of embryo development. Tree Genet. Genomes.

[97] Quintana-Escobar A.O., Nic-Can G.I., Galaz Avalos R.M., Loyola-Vargas V.M., Gongora-Castillo E. (2019). Transcriptome analysis of the induction of somatic embryogenesis in Coffea canephora and the participation of ARF and Aux/IAA genes. PeerJ.

[98] Juárez-González V.T., López-Ruiz B.A., Baldrich P., Luján-Soto E., Meyers B.C., Dinkova T.D. (2019). The explant developmental stage profoundly impacts small RNA-mediated regulation at the dedifferentiation step of maize somatic embryogenesis. Sci. Rep..

[99] Xu X., Chen X., Chen Y., Zhang Q., Su L., Chen X., Chen Y., Zhang Z., Lin Y., Lai Z. (2020). Genome-wide identification of miRNAs and their targets during early somatic embryogenesis in Dimocarpus longan Lour. Sci. Rep..

[100] Zhu C., Perry S.E. (2005). Control of expression and autoregulation of AGL15, a member of the MADS-box family. Plant J..

[101] Muday G.K., Rahman A., Binder B.M. (2012). Auxin and ethylene: Collaborators or competitors?. Trends Plant. Sci..

[102] Van de Poel B., Smet D., Van Der Straeten D. (2015). Ethylene and hormonal cross talk in vegetative growth and development. Plant. Physiol..

[103] Hu Y., Vandenbussche F., Van Der Straeten D. (2017). Regulation of seedling growth by ethylene and the ethylene–auxin crosstalk. Planta.

[104] Bai B., Su Y.H., Yuan J., Zhang X.S. (2013). Induction of somatic embryos in arabidopsis requires local YUCCA expression mediated by the down-regulation of ethylene biosynthesis. Mol. Plant.

[105] Harding E.W. (2003). Expression and Maintenance of Embryogenic Potential Is Enhanced through Constitutive Expression of AGAMOUS-Like 15. Plant. Physiol..

[106] Ledwoń A., Gaj M.D. (2011). LEAFY COTYLEDON1, FUSCA3 expression and auxin treatment in relation to somatic embryogenesis induction in Arabidopsis. Plant. Growth Regul..

[107] Tsuwamoto R., Yokoi S., Takahata Y. (2010). Arabidopsis EMBRYOMAKER encoding an AP2 domain transcription factor plays a key role in developmental change from vegetative to embryonic phase. Plant. Mol. Biol..

[108] Wójcik A.M., Nodine M.D., Gaj M.D. (2017). MiR160 and miR166/165 contribute to the LEC2-mediated auxin response involved in the somatic embryogenesis induction in arabidopsis. Front. Plant. Sci..

[109] Szyrajew K., Bielewicz D., Dolata J., Wójcik A.M. (2017). MicroRNAs Are Intensively Regulated during Induction of Somatic Embryogenesis in Arabidopsis. Front. Plant. Sci..

[110] O’Malley R.C., Huang S.S.C., Song L., Lewsey M.G., Bartlett A., Nery J.R., Galli M., Gallavotti A., Ecker J.R. (2016). Erratum: Cistrome and Epicistrome Features Shape the Regulatory DNA Landscape. Cell.

[111] Weijers D., Wagner D. (2016). Transcriptional Responses to the Auxin Hormone. Annu. Rev. Plant. Biol..

[112] Berdasco M., Alcázar R., García-Ortiz M.V., Ballestar E., Fernández A.F., Roldán-Arjona T., Tiburcio A.F., Altabella T., Buisine N., Quesneville H. (2008). Promoter DNA hypermethylation and gene repression in undifferentiated arabidopsis cells. PLoS ONE.

[113] Gao Y.R., Sun J.C., Sun Z.L., Xing Y., Zhang Q., Fang K.F., Cao Q.Q., Qin L. (2020). The MADS-box transcription factor CmAGL11 modulates somatic embryogenesis in Chinese chestnut (Castanea mollissima Blume). J. Integr. Agric..

[114] Li X., Wang X., He K., Ma Y., Su N., He H., Stolc V., Tongprasit W., Jin W., Jiang J. (2008). High-resolution mapping of epigenetic modifications of the rice genome uncovers interplay between DNA methylation, histone methylation, and gene expression. Plant. Cell.

[115] Zilberman D., Gehring M., Tran R.K., Ballinger T., Henikoff S. (2007). Genome-wide analysis of Arabidopsis thaliana DNA methylation uncovers an interdependence between methylation and transcription. Nat. Genet..

[116] To T.K., Saze H., Kakutani T. (2015). DNA methylation within transcribed regions. Plant. Physiol..

[117] Li W., Liu H., Cheng Z.J., Su Y.H., Han H.N., Zhang Y., Zhang X.S. (2011). DNA methylation and histone modifications regulate de novo shoot regeneration in arabidopsis by modulating wuschel expression and auxin signaling. PLoS Genet..

[118] Bräutigam K., Cronk Q. (2018). DNA methylation and the evolution of developmental complexity in plants. Front. Plant. Sci..

[119] Aliaga B., Bulla I., Mouahid G., Duval D., Grunau C. (2019). Universality of the DNA methylation codes in Eucaryotes. Sci. Rep..

[120] Boba A., Kostyn K., Preisner M., Wojtasik W., Szopa J., Kulma A. (2018). Expression of heterologous lycopene β-cyclase gene in flax can cause silencing of its endogenous counterpart by changes in gene-body methylation and in ABA homeostasis mechanism. Plant. Physiol. Biochem..

[121] Schmitz R.J., He Y., Valdés-López O., Khan S.M., Joshi T., Urich M.A., Nery J.R., Diers B., Xu D., Stacey G. (2013). Epigenome-wide inheritance of cytosine methylation variants in a recombinant inbred population. Genome Res..

[122] Dubin M.J., Zhang P., Meng D., Remigereau M.S., Osborne E.J., Casale F.P., Drewe P., Kahles A., Jean G., Vilhjálmsson B. (2015). DNA methylation in Arabidopsis has a genetic basis and shows evidence of local adaptation. Elife.

[123] Bewick A.J., Ji L., Niederhuth C.E., Willing E.M., Hofmeister B.T., Shi X., Wang L., Lu Z., Rohr N.A., Hartwig B. (2016). On the origin and evolutionary consequences of gene body DNA methylation. Proc. Natl. Acad. Sci. USA.

[124] Cerruti E., Comino C., Acquadro A., Marconi G., Repetto A.M., Pisanu A.B., Pilia R., Albertini E., Portis E. (2019). Analysis of DNA Methylation patterns associated with in vitro propagated Globe Artichoke plants using an EpiRADseq-Based Approach. Genes.

[125] Brenet F., Moh M., Funk P., Feierstein E., Viale A.J., Socci N.D., Scandura J.M. (2011). DNA methylation of the first exon is tightly linked to transcriptional silencing. PLoS ONE.

[126] Song K., Li L., Zhang G. (2017). The association between DNA methylation and exon expression in the Pacific oyster Crassostrea gigas. PLoS ONE.

[127] Liang L., Chang Y., Lu J., Wu X., Liu Q., Zhang W., Su X., Zhang B. (2019). Global methylomic and transcriptomic analyses reveal the broad participation of DNA methylation in daily gene expression regulation of Populus trichocarpa. Front. Plant. Sci..

[128] Ball M.P., Li J.B., Gao Y., Lee J.H., Leproust E.M., Park I.H., Xie B., Daley G.Q., Church G.M. (2009). Targeted and genome-scale strategies reveal gene-body methylation signatures in human cells. Nat. Biotechnol..

[129] Chodavarapu R.K., Feng S., Bernatavichute Y.V., Chen P.Y., Stroud H., Yu Y., Hetzel J.A., Kuo F., Kim J., Cokus S.J. (2010). Relationship between nucleosome positioning and DNA methylation. Nature.

[130] Anastasiadi D., Esteve-Codina A., Piferrer F. (2018). Consistent inverse correlation between DNA methylation of the first intron and gene expression across tissues and species. Epigenetics Chromatin.

[131] Wang X., Duan C.G., Tang K., Wang B., Zhang H., Lei M., Lu K., Mangrauthia S.K., Wang P., Zhu G. (2013). RNA-binding protein regulates plant DNA methylation by controlling mRNA processing at the intronic heterochromatin-containing gene IBM1. Proc. Natl. Acad. Sci. USA.

[132] Ong-Abdullah M., Ordway J.M., Jiang N., Ooi S.E., Kok S.Y., Sarpan N., Azimi N., Hashim A.T., Ishak Z., Rosli S.K. (2015). Loss of Karma transposon methylation underlies the mantled somaclonal variant of oil palm. Nature.

[133] Deng S., Chua N.H. (2015). Inverted-Repeat RNAs Targeting FT Intronic Regions Promote FT Expression in Arabidopsis. Plant. Cell Physiol..

[134] EI Baidouri M., Kim K.D., Abernathy B., Li Y.H., Qiu L.J., Jackson S.A. (2018). Genic C–methylation in soybean is associated with gene paralogs relocated to transposable element-rich pericentromeres. Mol. Plant.

[135] Takahashi S., Osabe K., Fukushima N., Takuno S., Miyaji N., Shimizu M., Takasaki-Yasuda T., Suzuki Y., Dennis E.S., Seki M. (2018). Genome-wide characterization of DNA methylation, small RNA expression, and histone H3 lysine nine di-methylation in *Brassica rapa* L.. DNA Res..

[136] Yang H., Chang F., You C., Cui J., Zhu G., Wang L., Zheng Y., Qi J., Ma H. (2015). Whole-gemone DNA methylation patterns and complex associations with gene structure and expression during flower development in Arabidopsis. Plant J..

[137] Wang X., Hu L., Wang X., Li N., Xu C., Gong L., Liu B. (2016). DNA Methylation Affects Gene Alternative Splicing in Plants: An Example from Rice. Mol. Plant.

[138] Le T.N., Miyazaki Y., Takuno S., Saze H. (2015). Epigenetic regulation of intragenic transposable elements impacts gene transcription in Arabidopsis thaliana. Nucleic Acids Res..

[139] Espinas N.A., Tu L.N., Furci L., Shimajiri Y., Harukawa Y., Miura S., Takuno S., Saze H. (2020). Transcriptional regulation of genes bearing intronic heterochromatin in the rice genome. PLoS Genet..

[140] Ullah F., Hamilton M., Reddy A.S.N., Ben-Hur A. (2018). Exploring the relationship between intron retention and chromatin accessibility in plants. BMC Genom..

[141] Deremetz A., Le Roux C., Idir Y., Brousse C., Agorio A., Gy I., Parker J.E., Bouché N. (2019). Antagonistic actions of FPA and IBM2 regulate transcript processing from genes containing heterochromatin. Plant Physiol..

[142] Gamborg O.L., Miller R.A., Ojima K. (1968). Nutrient requirements of suspension cultures of soybean root cells. Exp. Cell Res..

[143] Thellin O., Zorzi W., Lakaye B., De Borman B., Coumans B., Hennen G., Grisar T., Igout A., Heinen E. (1999). Housekeeping genes as internal standards: Use and limits. J. Biotechnol..

[144] Ledwoń A., Gaj M.D. (2009). LEAFY COTYLEDON2 gene expression and auxin treatment in relation to embryogenic capacity of Arabidopsis somatic cells. Plant. Cell Rep..

[145] Gliwicka M., Nowak K., Ciela E., Gaj M.D. (2012). Expression of seed storage product genes (CRA1 and OLEO4) in embryogenic cultures of somatic tissues of Arabidopsis. Plant. Cell. Tissue Organ. Cult..

[146] Nowak K., Wojcikowska B., Gaj M.D. (2015). ERF022 impacts the induction of somatic embryogenesis in Arabidopsis through the ethylene-related pathway. Planta.

[147] Doyle J.J., Doyle J.L. (1987). A rapid DNA isolation procedure for small quantities of fresh leaf tissue. Phytochem. Bull..

[148] Fouse S.D., Nagarajan R., Costello J.F. (2010). Genomic analysis of DNA methylation. Epigenomics.

[149] Du J., Johnson L.M., Jacobsen S.E., Dinshaw J.P. (2015). DNA methylation pathways and their crosstalk with a histone methylation. Natl. Rev. Malecular Cell Biol..

[150] Mozgova I., Köhler C., Hennig L. (2015). Keeping the gate closed: Functions of the polycomb repressive complex PRC2 in development. Plant J..

[151] Mozgová I., Muñoz-Viana R., Hennig L. (2017). PRC2 Represses Hormone-Induced Somatic Embryogenesis in Vegetative Tissue of Arabidopsis thaliana. PLoS Genet..

[152] Peng M., Cui Y., Bi Y.-M., Rothstein S.J. (2006). AtMBD9: A protein with a methyl-CpG-binding domain regulates flowering time and shoot branching in Arabidopsis. Plant J..

[153] Yaish M.W., Peng M., Rothstein S.J. (2009). AtMBD9 medulates Arabidopsis development through the dual epigenetic pathways of DNA methylation and histone acetylation. Plant J..

[154] Wójcikowska B., Botor M., Morończyk J., Wójcik A.M., Nodzyński T., Karcz J., Gaj M.D. (2018). Trichostatin a triggers an embryogenic transition in arabidopsis explants via an auxin-related pathway. Front. Plant. Sci..

[155] Siddiqui Z.H., Abbas Z.K., Ansari M.W., Khan M.N. (2019). The role of miRNA in somatic embryogenesis. Genome.

[156] Wu L., Zhou H., Zhang Q., Zhang J., Ni F., Liu C., Qi Y. (2010). DNA Methylation Mediated by a MicroRNA Pathway. Mol. Cell.

[157] Bao N., Lye K.W., Barton M.K. (2004). MicroRNA binding sites in Arabidopsis class III HD-ZIP mRNAs are required for methylation of the template chromosome. Dev. Cell.

[158] Zhao Y., Mo B., Chen X. (2012). Mechanisms that impact microRNA stability in plants. RNA Biol..

[159] Narsai R., Gouil Q., Secco D., Srivastava A., Karpievitch Y.V., Liew L.C., Lister R., Lewsey M.G., Whelan J. (2017). Extensive transcriptomic and epigenomic remodelling occurs during Arabidopsis thaliana germination. Genome Biol..

[160] Glaich O., Parikh S., Bell R.E., Mekahel K., Donyo M., Leader Y., Shayevitch R., Sheinboim D., Yannai S., Hollander D. (2019). DNA methylation directs microRNA biogenesis in mammalian cells. Nat. Commun..

