# Supplementary material for: Hypermethylation of Auxin-Responsive Motifs in the Promoters of the Transcription Factor Genes Accompanies the Somatic Embryogenesis Induction in Arabidopsis
Source: Int J Mol Sci. 2020 Sep 18;21(18):6849. doi: 10.3390/ijms21186849 (PMC7555384; doi:10.3390/ijms21186849)
Supplement: Supplementary file 1 [file ijms-21-06849-s001.pdf]

Table S1. Primers that were used to analyse gene expression (RTqPCR).

gene	accession number	Primers sequence (5′-3′)	Referencess
LEC2	AT1G28300	Forward =AGGGAAAGGAACCACTACGAA<br>Reverse = CAGTGGTGAGGTCCATGAGAT	[1]
LEC1	AT1G21970	Forward = AGAGACCGATCGTGGTTCTG<br>Reverse = GAAGAGCCACCACCAACACT	[2]
BBM	AT5G17430	Forward = AATGCTAATCATCAAGACAAT<br>Reverse = ATCTACCTGTCCACCGATGC	[2]
WUS	AT2G17950	Forward = TCACCATCATCACGGTGTTC<br>Reverse = AGAACAGTCTTGTTCCATAGA	-
AGL15	AT5G13790	Forward = CAAGGGCTTGAATCCTCTGA<br>Reverse = GTTGTTCCTTGAGGCGTGAT	-

Table S2. Characteristics of the regulatory motifs in the promoters of the SE-involved genes that were analysed using the magMeDIP method. The motifs include: the ARF-binding (consensus sequence: TGTCTC) and the ethylene-response-element (consensus sequence: TTTGAAAT) sequences (AGRIS, http://arabidopsis.med.ohio-state.edu/AtcisDB; PLANT CARE, http://bioinformatics.psb.ugent.be; SOFT BERRY, http://linux1.softberry.com/).

Gene (accession number)	Description (reference)	Position of analyzed promotor fragment	Regulatory elements within promotor fragments	Position of analyzed gene body fragment
LEC1 AT1G21970	Ectopic expression in vegetative cells induces the expression of embryo-specific genes and initiates formation of embryo-like structures [3]	-72 – -172 bp	3 x LFY-binding motif<br>1 x ARF-binding motif	157 – 268 bp (1st intron**)
LEC2 AT1G28300	Knock-out leads to strong impaired in SE induction response *in vitro* [4]	-25 – -237 bp	ARF-binding motif<br>cis-element that regulates trimethylation of H3K27*	115 – 236 bp (1st exon)
BBM AT5G17430	Ectopic expression in Arabidopsis and Brassica leads to the spontaneous formation of somatic embryos on seedlings [5]	-455 – -584 bp	ARF-binding motif	64 – 242 bp (1st exon)
WUS AT2G17950	Overexpression leads to formationg somatic embryos on hormone-free medium [6]	-497 – -650 bp	ARF-binding motif	186 – 380 bp (1st exon)
AGL15 AT5G13790	Overexpression enhacing SE in Arabidopsis and soybean. Knock-out disturbed SE induction by decreasing frequency of somatic embryo development [7]	-389 – -494 bp	Ethylene response element	70 – 210 bp (1st exon)

* H3K27 = Histone H3 at Lysine-27

** the *LEC1* gene has very short 1st exon, therefore due to the technical issue the analyzed 'gene body' fragment was choose in vicinity of this region

Table S3. The gene primer sequences that were selected for the magMeDIP analysis.

gene	accession number	Type of sequence	primers sequences
LEC2	AT1G28300	promotor	Forward = CTGGCGCCACATATACACAT<br>Reverse = AGAGAGGTCGTTGGGGAGAT
		gene body	Forward = GCTCGCACTTCACAACAGTC<br>Reverse = GTAAACCGGCTGAGGATTCA
LEC1	AT1G21970	promotor	Forward = AAAACAACCAATGAGAGAAGAGC<br>Reverse = GCAAAGGAGGCGGTTCTTAC
		intron	Forward = TCATGAAAAATTGGAGACATGC<br>Reverse = CCATTCCCAAAACCTTCACA
BBM	AT5G17430	promotor	Forward = AAAGTGACGAGTTCGCGTTT<br>Reverse = CCAAAAACTCACTCGTTGTCG
		gene body	Forward = ACGGATGTTGACTCCTCCAC<br>Reverse = AAACACAAACCTCGGGAGTG
WUS	AT2G17950	promotor	Forward = TTGTTTTGTTTTCTGTGTGTATGG<br>Reverse = GTGAGGGCATCATCGGTATT
		gene body	Forward = ACAACAACAAGTCCGGCTCT<br>Reverse = AAAAGACGTTCTTGCCCTCA
AGL15	AT5G13790	promotor	Forward = CATCAAACAATGCTAGTTGTTGC<br>Reverse = AACAATGTTGACCGACATAAAAA
		gene body	Forward = GGATCGAGAATGCGAATAGC<br>Reverse = ACTCGAAGAGCTTGCCAGAC

Table S4. Methyl DNA enrichment (%) in the promotor (P) fragments of the SE-involved TF genes on different 2,4-D concentrations (0.0 μM; 0.1 μM; 5.0 μM; 20.0 μM). Analysis was performed using the magMeDIP method and the percentage of P fragments with methylated cytosine were evaluated. Values that were significantly different than 0d are indicated with an asterisk (*) (Student's t test, $p < 0.05$).

Gene	2,4-D concentration in medium (μM)	Methyl DNA enrichment (%) in promotor fragments (P) of analyzed genes at selected time points of culture			
		0 d	3 d	5 d	10 d
LEC1	0.0	17.89 +/- 0.35	2.66* +/- 0.34	5.80* +/- 1.03	8.31* +/- 1.02
	0.1		9.13* +/- 1.17	8.08* +/- 1.02	19,70* +/- 0.98
	5.0		23.40* +/- 1.99	21.70* +/- 0.51	15,10* +/- 0.63
	20.0		6.30* +/- 0.64	9.35* +/- 0.66	8,34* +/- 1.05
LEC2	0.0	18.66 +/- 0.86	1.04* +/- 0.32	2.62* +/- 0.46	3.99* +/- 0.20
	0.1		2.30*+/- 0.31	3.39* +/- 0.63	4.63* +/- 0.71
	5.0		10.92* +/- 1.25	13.54* +/- 0.60	6.71* +/- 1.38
	20.0		2.18* +/- 0.45	3.62* +/- 0.47	3.06* +/- 0.20
BBM	0.0	4.52 +/- 0.47	0.51* +/- 0.10	1.01* +/- 0.13	2.30* +/- 0.45
	0.1		0.84* +/- 0.16	2.24* +/- 0.39	1.96* +/- 0.36
	5.0		4.39 +/- 0.14	2.38* +/- 0.26	2.64* +/- 0.16
	20.0		0.79* +/- 0.34	1.27* +/- 0.26	1.04* +/- 0.40
WUS	0.0	14.42 +/- 2.66	0.49* +/- 0.13	1.40* +/- 0.24	8.20 +/- 1.36
	0.1		1.25* +/- 0.23	1.07* +/- 0.13	2.31* +/- 0.10
	5.0		8.00* +/- 0.87	5.76* +/- 0.11	3.96* +/- 0.86
	20.0		0.96* +/- 0.27	1.24* +/- 0.33	1.04* +/- 0.51
AGL15	0.0	10.99 +/- 1.89	0.27* +/- 0.07	0.74* +/- 0.09	1.99* +/- 0.11
	0.1		0.79* +/- 0.16	0.81* +/- 0.06	2.67* +/- 0.06
	5.0		10.27 +/- 0.07	11.30 +/- 2.27	8.71 +/- 1.54
	20.0		0.54* +/- 0.05	1.28* +/- 0.17	0.76* +/- 0.06

Table S5. Methyl DNA enrichment in the gene body (GB) fragments of the SE-involved TF genes on different 2,4-D concentrations (0.0 μM; 0.1 μM; 5.0 μM; 20.0 μM) Analysis was performed using the magMeDIP method and the percentage of the GB fragments with methylated cytosine were evaluated. Values significantly different from the 0 d are indicated with an asterisk (*) (Student's t test, $p < 0.05$).

**Gene**	**2,4-D concentration in medium (μM)**	**Methyl DNA enrichment (%) in gene body fragments (GB) of analyzed genes at selected time points of culture**			
		**0 d**	**3 d**	**5 d**	**10 d**
LEC1	0.0	12.82 +/- 2.66	3.62* +/- 0,40	10.36 +/- 1,35	12.63 +/- 0.66
	0.1		4.10* +/- 0,76	1.69* +/- 0,13	4.18* +/- 0.69
	5.0		1.77* +/- 0,09	1.85* +/- 0,08	2.11* +/- 0.41
	20.0		1.38* +/- 0,20	1.92* +/- 0,26	4.03* +/- 0.65
LEC2	0.0	2.36 +/- 0.30	1.70* +/- 0.18	1.89 +/- 0.26	4.40* +/- 0.07
	0.1		1.37* +/- 0.21	1.61 +/- 0.54	1.62* +/- 0.09
	5.0		0.77* +/- 0.02	0.75* +/- 0.20	1.75 +/- 0.40
	20.0		5.10* +/- 0.40	4.59* +/- 0.60	2.28 +/- 0.42
BBM	0,0	1.19 +/- 0.08	1,40 +/- 0.24	1.44 +/- 0.23	1.86* +/- 0.12
	0,1		1.54* +/- 0.10	1.41 +/- 0.38	2.55* +/- 0.30
	5,0		0.54* +/- 0.11	1.14 +/- 0.12	0.38* +/- 0.13
	0.0		6.49* +/- 0.33	5.54* +/- 0.51	1.67* +/- 0.23
WUS	0.0	2.92 +/- 0.70	0.31* +/- 0.13	0.76* +/- 0.02	1.22* +/- 0.44
	0.1		0.68* +/- 0.09	1.27* +/- 0.06	0.75* +/- 0.06
	5.0		0.76* +/- 0.06	1.63* +/- 0.29	0.34* +/- 0.13
	20.0		3.14 +/- 0.33	2.51 +/- 0.51	2.55 +/- 0.39
AGL15	0.0	2,57 +/- 0,25	0.86* +/- 0.05	0.67* +/- 0.02	1.05* +/- 0.05
	0.1		1.88* +/- 0.20	2.10 +/- 0.61	3.12 +/- 0.60
	5.0		1.76* +/- 0.06	1.21* +/- 0.06	0.79* +/- 0.13
	20.0		6.90* +/- 0.72	5.27* +/- 0.44	3.99* +/- 0.38

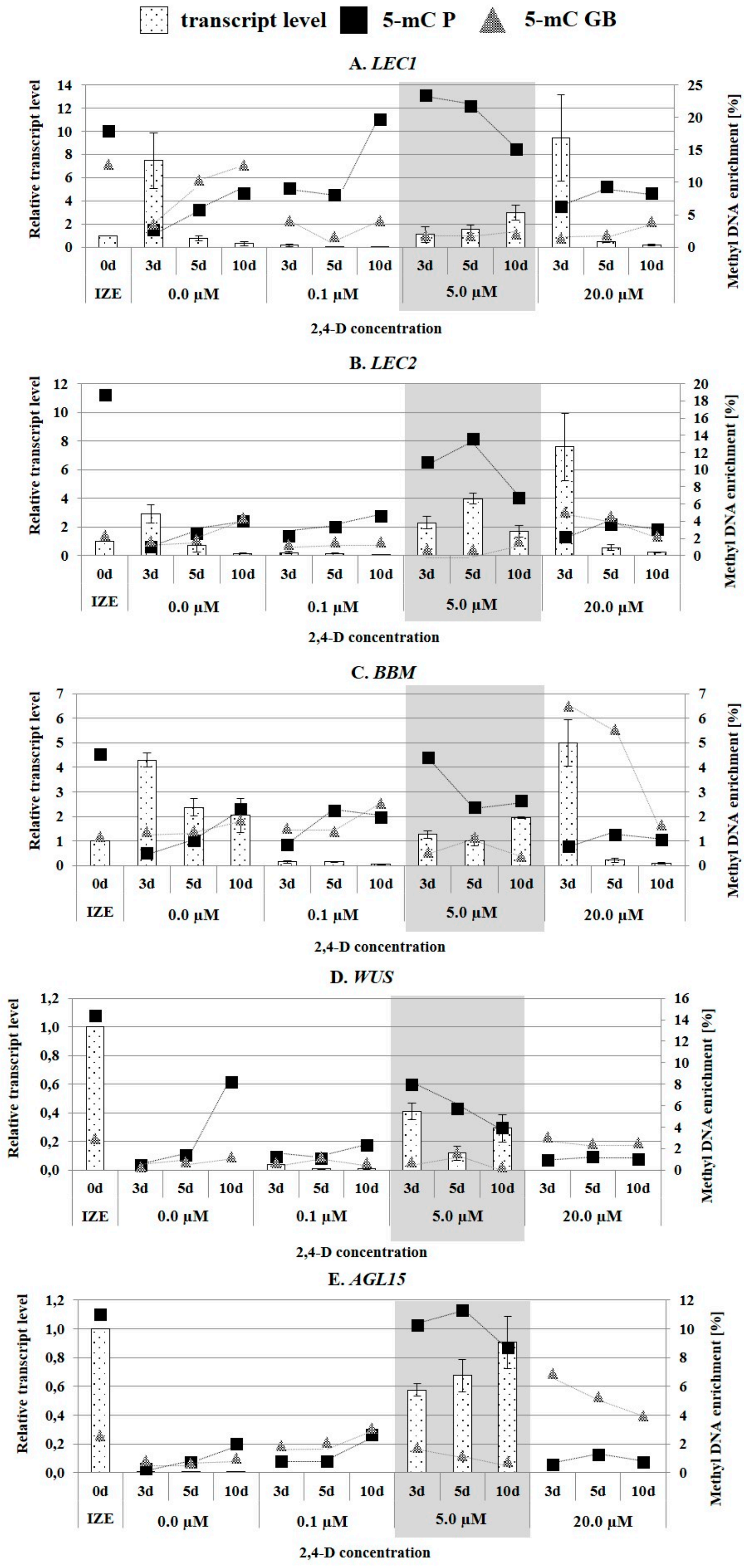

Figure S1. Transcript level of the SE-involved *TF* genes (**A-E**) relative to the methylation of promoter (P) and the gene body (GB) in explants that were cultured on media with different 2,4-D concentrations (0.0 µM; 0.1 µM; 5.0 µM; 20.0 µM). A. *LEC1*, B. *LEC2*, C. *BBM*, D. *WUS*, E. *AGL15*. The analysed GB fragments include the intron (*LEC1*) and exon (*LEC2*, *BBM*, *WUS*, *AGL15*) regions. Error bars indicate the standard deviation (SD).

Reference

1. Ledwoń, A.; Gaj, M.D. LEAFY COTYLEDON2 gene expression and auxin treatment in relation to embryogenic capacity of Arabidopsis somatic cells. *Plant Cell Rep.* **2009**, *28*, 1677–1688, doi:10.1007/s00299-009-0767-2.
2. Grzybkowska, D.; Morończyk, J.; Wójcikowska, B.; Gaj, M.D. Azacitidine (5-AzaC)-treatment and mutations in DNA methylase genes affect embryogenic response and expression of the genes that are involved in somatic embryogenesis in Arabidopsis. *Plant Growth Regul.* **2018**, *85*, 243–256, doi:10.1007/s10725-018-0389-1.
3. Lotan, T.; Ohto, M.; Yee, K.M.; West, M.A; Lo, R.; Kwong, R.W.; Yamagishi, K.; Fischer, R.L.; Goldberg, R.B.; Harada, J.J. Arabidopsis leafy cotyledon1 is sufficient to induce embryo development in vegetative cells. *Cell* **1998**, *93*, 1195–1205. doi: 10.1016/S0092-8674(00)81463-4.
4. Gaj, M.D.; Zhang, S.; Harada, J.J.; Lemaux, P.G. Leafy cotyledon genes are essential for induction of somatic embryogenesis of Arabidopsis. *Planta.* **2005**, *222*, 977–988. doi: 10.1007/s00425-005-0041-y.
5. Boutilier, K. Ectopic Expression of BABY BOOM Triggers a Conversion from Vegetative to Embryonic Growth. *Plant Cell* **2002**, *14*, 1737–1749. doi: 10.1105/tpc.001941.tissue.
6. Zuo, J.; Niu, Q.W.; Frugis, G.; Chua, N.H. The WUSCHEL gene promotes vegetative-to-embryonic transition in Arabidopsis. *Plant., J.* **2002**, *30*, 349–359. doi: 10.1046/j.1365-313X.2002.01289.x.
7. Thakare, D.; Tang, W.; Hill, K.; Perry, S.E. The MADS-Domain Transcriptional Regulator agamous – like15 Promotes Somatic Embryo Development in Arabidopsis and Soybean. *Plant. Physiol.* **2008**, *146*, 1663–1672. doi: 10.1104/pp.108.115832.
